# Regulation of Neuroendocrine-like Differentiation in Prostate Cancer by Non-Coding RNAs

**DOI:** 10.3390/ncrna7040075

**Published:** 2021-12-02

**Authors:** Eva Slabáková, Zuzana Kahounová, Jiřina Procházková, Karel Souček

**Affiliations:** Department of Cytokinetics, Institute of Biophysics of the Czech Academy of Sciences, 61265 Brno, Czech Republic; slabakova@ibp.cz (E.S.); pernicova@ibp.cz (Z.K.); prochazkova@ibp.cz (J.P.)

**Keywords:** neuroendocrine differentiation/transdifferentiation, microRNA, lncRNA, prostate cancer, patients’ dataset, liquid biomarkers, exosomes, extracellular vesicles

## Abstract

Neuroendocrine prostate cancer (NEPC) represents a variant of prostate cancer that occurs in response to treatment resistance or, to a much lesser extent, *de novo*. Unravelling the molecular mechanisms behind transdifferentiation of cancer cells to neuroendocrine-like cancer cells is essential for development of new treatment opportunities. This review focuses on summarizing the role of small molecules, predominantly microRNAs, in this phenomenon. A published literature search was performed to identify microRNAs, which are reported and experimentally validated to modulate neuroendocrine markers and/or regulators and to affect the complex neuroendocrine phenotype. Next, available patients’ expression datasets were surveyed to identify deregulated microRNAs, and their effect on NEPC and prostate cancer progression is summarized. Finally, possibilities of miRNA detection and quantification in body fluids of prostate cancer patients and their possible use as liquid biopsy in prostate cancer monitoring are discussed. All the addressed clinical and experimental contexts point to an association of NEPC with upregulation of miR-375 and downregulation of miR-34a and miR-19b-3p. Together, this review provides an overview of different roles of non-coding RNAs in the emergence of neuroendocrine prostate cancer.

## 1. Introduction

Neuroendocrine prostate cancer (NEPC) is a highly aggressive treatment-resistant stage of prostate cancer (PCa) with poor patient outcome. It often occurs after long-term androgen-deprivation therapy; however, *de novo* pure neuroendocrine tumours are also described. Prostate cancer cells undergo robust phenotypic changes (so called transdifferentiation), resulting in the neuroendocrine-like phenotype. This is associated with low or absent signalling of the androgen receptor (AR), neuron-like morphology, expression and secretion of neuropeptides and biologically active factors, and deregulation of expression of several molecular drivers (e.g., Rb, TP53, MYCN) [1]. The molecular machinery behind the development of NEPC is still being investigated, as well as the elucidation of the role of microRNAs in the induction and regulation of NEPC. So far, deregulation of several oncogenic miRNAs (e.g., miR-21, miR-141, miR-32, miR-650, miR-106b/miR-25 cluster, and others) as well as tumour suppressor miRNAs (e.g., miR-34, miR-145, miR-200b, let-7 miRNAs, and others) was described in the context of PCa (summarized in [2]). Moreover, miRNAs are also investigated as potential biomarkers for PCa diagnosis. Interestingly, one molecule can exert the opposite action in different contexts. Namely, miR-204 acts as a tumour suppressor in prostate cancer cells and as an oncomiR in neuroendocrine cancer cells [3]. Similarly, LINC00261 was described as a tumour suppressor in multiple cancers [4], while promoting tumorigenesis in prostate neuroendocrine cells [5]. Therefore, a deep understanding of the involvement of particular miRNAs in NEPC emergence and pathology is desirable. This review article summarizes the findings concerning potential miRNA and other non-coding RNA regulators in the context of neuroendocrine transdifferentiation of advanced prostate cancer.

### Neuroendocrine Prostate Cancer

Prostate cancer (PCa) is the second most often diagnosed and fifth leading cause of cancer death among men worldwide [6]. Primary therapy of clinically localized non-metastatic disease consists of radical prostatectomy and/or radiotherapy [7]. Since the growth and function of the prostate gland as well as cancer cells are dependent on the action of steroid hormones androgens, targeting androgen production or signalling of AR is used for treatment of recurrent and metastatic PCa. Although initially responding to androgen deprivation therapy (ADT), cancer cells can adapt to ADT and restore AR signalling under low levels of androgens, and consequently, the disease progresses to more aggressive castration-resistant prostate cancer (CRPC) [8]. Since CRPC is still dependent on AR signalling, next-generation AR pathway inhibitors (ARPIs) are used to inhibit intratumoral androgen biosynthesis (abiraterone acetate) or block AR function (enzalutamide) with significant clinical benefits [9]. However, in about 20–25% of patients, resistance may also develop to these newer agents following ADT. Several mechanisms of resistance were described as a consequence of the restoration of AR signalling—gain-of-function mutations of AR, upregulation of the constitutive active AR splice variant, increased intratumoral androgen biosynthesis, or bypassing AR signalling by signalling of glucocorticoid receptor (GR) (summarized in [8,10]). Prolonged androgen inhibition treatment leads to progression to the advanced stage of CRPC associated with reversible transdifferentiation of cancer cells, which lose prostate cell characteristics and acquire neuroendocrine characteristics. This highly aggressive stage with rapid tumour dissemination and therapy resistance is called treatment-emergent neuroendocrine prostate cancer (t-NEPC) (summarized in [1,11]).

The prostate epithelium is composed of basal, luminal, and neuroendocrine cells (NE cells). NE cells are the least abundant cell type in prostate epithelium and are scattered among basal and luminal cells. The origin of NE cells is still ambiguous; NE cells are either derived from common prostate stem cell (summarized in [12]) or they migrate from the neural crest to the glandular prostate epithelium [13]. It is assumed that NE cells are involved in the growth and differentiation of the prostate as well as in the regulation of secretion of the prostatic gland [14]. NE cells do not express AR or the prostate specific antigen (PSA) and are postmitotic [15]. These cells contain and secrete a variety of factors and neuropeptides, which can act in an endocrine, autocrine, and paracrine manner on target cells, as discussed subsequently (summarized in [16,17]). NE cells are found scattered also in prostate adenocarcinoma with a similar frequency to normal prostate epithelium (no more than 1%) (reviewed in [17]). However, in advanced stages (metastatic CRPC, mCRPC) an increasing number of foci of cells with NE characteristics are found.

NE cells and NE-like cancer cells express and secrete a broad spectrum of biological active factors and neuropeptides, denominated together as NED markers. Some of these markers are also detected in patients’ blood/serum/plasma, which makes them useful biomarkers of cancer progression. Importantly, both NE cells and surrounding cancer cells express several receptors for these factors; therefore, these factors can act in an autocrine or paracrine manner and can support the growth of both cell types (summarized in [18,19]). For detailed characterisation and role of NED markers in PCa and/or NEPC, see Table 1.

*De novo* pure neuroendocrine tumours, so called small cell carcinomas (SCC), are a very rare (0.5–2.0%) and very aggressive subtype of NEPC. They are characterized by low PSA levels, short or no response to conventional ADT, and the presence of lytic bone metastasis and intracranial metastasis (summarized in [93]). A total of 94% of SCCs were positive for at least one NE marker (CgA, NSE, Syp, CD56) [94]. Up to 50% of men diagnosed with SSC have a history of conventional prostatic carcinoma [93]. More often, cancer cells with similar characteristics to SCC mixed with adenocarcinoma are found. This is frequently found in patients progressing after ADT [95]. NEPC is characterized by low or absent AR signalling, loss of RB1 and TP53, amplification of MYCN, ERG rearrangement, upregulation of BRN2, down-regulation of DNA methyltransferases and altered DNA methylation, and upregulation of EZH2 and Polycomb-mediated gene silencing. These tumours are positive for NED markers CgA, NSE, SYP, or CD56 and negative for luminal markers (PSA and PAP). Patients with NEPC are treated with platinum-based chemotherapy, and the survival ranges from 7 months to 2 years (summarized in [1,10]).

The origin of NEPC is still ambiguous. The possible mechanisms leading to NED induction are followed: AR-targeted therapies, other therapies (cyclooxygenase-2 inhibitors, genistein, ionizing radiation), various cells from tumour microenvironment (cancer-associated fibroblasts, mast cells, macrophages, bone marrow-derived cells), Ca^2+^ ion channels and Ca^2+^ ion homeostasis, or exosomes [1]. Recent studies show that the lineage plasticity (transition from one developmental pathway to another) also plays a role in the context of NEPC development and therapy resistance. Specifically, the lineage plasticity is associated with the acquisition of independence on AR signalling and treatment resistance in about 20% of advanced PCa patients. This progressive state of CRPC is associated with the loss of AR-regulated lineage characteristics (luminal epithelial phenotype) and, in some situations, the acquisition of new phenotypes (e.g., NE features, NEPC), with involvement of metabolic, genetic, and epigenetic changes (summarized in [96]). There is evidence that NE-like cancer cells in CRPC arise through transdifferentiation from luminal epithelial cells in the mouse CRPC model [97]. Recently, Dong and colleagues proposed a model of PCa development, where NE-like cancer cells arise through transdifferentiation of luminal cancer cells, and these NE-like cancer cells are responsible for at first focal NED, which evolves in pure NEPC [98]. Nouri and colleagues showed that, in response to ARPIs, androgen-sensitive PCa cells are reprogrammed to cancer stem-like cells with characteristics of metastable neural/neural crest stem cells, which can transdifferentiate in neuroendocrine-like PCa cells [99]. Importantly, since the neural crest-derived origin of major fraction of normal NE cells in both human and mouse prostate was experimentally described [13], the possible origin of NE-like cancer cells from these neural crest-derived NE cells should be also taken into account and examined. Further research is needed to understand the evolution of CRPC and NEPC and the molecular machinery behind this to be able to develop potent treatment strategies.

## 2. Regulatory Circuits Driving Neuroendocrine Differentiation in Prostate Cancer

During the acquisition of neuroendocrine phenotype, prostate cancer cells have been reported to undergo complex remodelling of their transcriptional and phenotypical landscapes (reviewed in, e.g., [100,101,102]). The list of underlying molecular mechanisms growths year by year [103,104] and also involves processes such as enhanced infiltration of the primary tumour with early neural progenitors or direct interaction of cancer cells with nerves present in the reshaping tumour microenvironment [105,106,107]. Since the complexity of such regulatory circuits is multispectral and still emerges, here, we briefly preferentially summarize those driving events that were experimentally validated in *in vivo* and/or *in vitro* studies and are represented by deregulated functions of specific tumour suppressors, oncogenes, and transcription factors (TFs) in prostate cancer cells.

### 2.1. Signalling and Genetic Hallmarks of mCRPC Samples

Integrative genomic analysis of mCRPC samples revealed an accumulation of multiple somatic aberrations in genes encoding *AR* (amplification) and tumour suppressors *p53, PTEN*, and *RB1* (deletions and/or mutations) [108]. These genetic perturbations support genomic instability, cancer cell survival, dedifferentiation, and pro-neuronal differentiation [109,110,111]. The complexity and type of changes that lead to the deregulation of AR, PTEN, p53, and pRB signalling during PCa progression is a matter of intensive investigation and vivid discussions [112,113,114,115,116]. They may encompass processes such as expression of constitutively active AR variants [117], ligand-independent activation of AR [118], or expression of gain-of-function p53 mutants, which have been recently demonstrated to induce conversion of fibroblasts to a cancer-associated phenotype that supports increased tumour growth and metastasis [108,119,120]. Acquisition of the neuroendocrine program is accompanied by genetic alterations and rewiring of other important signalling networks such as those mediated by Aurora kinase A (AURKA) or PI3K/AKT [121,122,123,124].

### 2.2. ‘Lost and Found’ Protein Keys Unlocking Neuroendocrine Trans-Differentiation of Prostate Cancer

EHF (also known as ESE3) is an epithelial-specific ETS transcription factor previously shown to be highly expressed in normal prostate tissue, where it prevents prostate pathogenesis and contributes to the maintenance of homeostasis and differentiation status of epithelial cells. EHF expression is reduced in PCa samples, and its re-expression inhibits the clonogenic survival of PCa cells and promotes their apoptosis [125]. EHF deficiency or loss induces an epithelial-to-mesenchymal transition (EMT) and endows epithelial prostate cells with stem-like features and tumour-initiating and metastatic properties [126]. Recently, EHF loss has been demonstrated to facilitate the development of treatment-induced NEPC via transcriptional de-repression of EZH2 and LIN28B and consequential deregulation of let-7 miRNAs expression and its maturation [127,128,129].

A similar impact on NEPC development is caused by the RE1 silencing transcription factor (REST), which is known as a transcriptional repressor of neuronal genes in neural progenitors and in non-neuronal tissue including prostate [111,130,131]. Loss of REST activity mediated by a splicing regulator serine/arginine repetitive matrix 4 (SRRM4) has been suggested to promote the emergence of the NE phenotype in CRPC [111] and endows cancer cells with stemness and neuroendocrine features most likely by de-repressing expression of REST targets such as CD44, Twist1, and secretagogin (SCGN) [131,132].

Finally, a transcription factor forkhead box A1 (FOXA1), previously reported due to its pioneering chromatin remodelling activity, which mediates an access of various nuclear receptors to their target regulatory regions, is an internal component of transcriptional program controlling AR signalling status [101,133]. FOXA1 co-regulates an AR-mediated transcriptional program in healthy prostate and in primary tumours via making response elements (ARE) accessible to AR and/or by direct interaction with AR itself. Additionally, in cooperation with other TFs, FOXA1 facilitates the oncogenic switch of AR signalling, yet its expression has a tumour-suppressive impact on the progression of primary PCa to NEPC [134,135,136,137].

Generally, TFs involved in nervous system development, especially those engaged in the transcriptional control of early neurogenesis, play essential roles in the process of prostate cancer neuroendocrine differentiation [138,139,140,141,142,143,144,145]. Sex-determining region Y2 (SOX2) [146], Achaete-scute family BHLH transcription factor 1 (ASCL1; also known as hASH1 or Mash1) [147,148,149], Doublecortin (DCX) [106,150], Forkhead box A2 (FOXA2) [151,152], POU class 3 homeobox 2 and 4 (POU3F2 and 4 also known as BRN2 and 4) [153,154], Neuroblastoma MYC oncogene (MYCN) [121,122,155,156], and One cut homeobox 2 (ONECUT2) [157] have been all demonstrated as active components of NEPC development and represent functional molecular tools essential for the reshaping of prostate epithelial tumours into neuroendocrine [98]. Similarly, remodelling of epigenetic and transcriptional landscapes mediated by transcriptional repressor Enhancer of Zeste homolog 2 (EZH2) [122,158,159], pro-neuronal splicing regulator SRRM4 [160,161], or transposable element Paternally expressed 10 (PEG10) [162,163] has been reported to promote neuroendocrine differentiation of PCa as well.

### 2.3. Rewiring, Remodelling, and Reshaping—A Vicious Program Turned on

**Figure 1:**
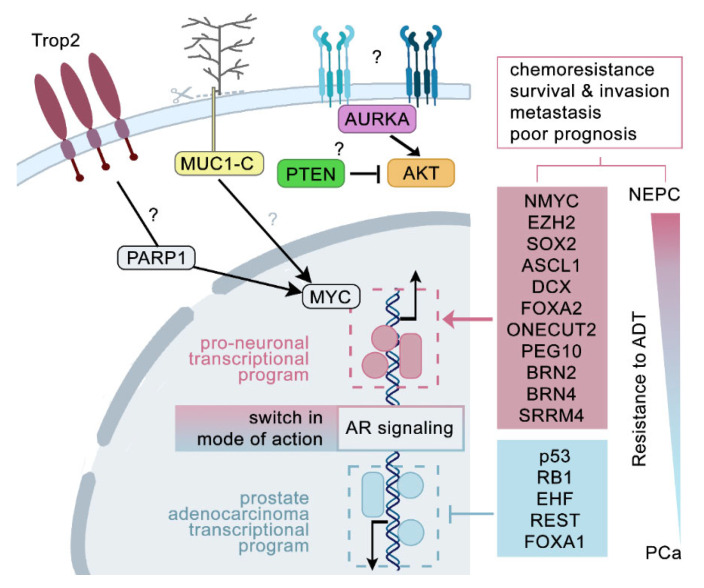
Components of regulatory circuits and driving events involved in NEPC development.

The rewiring of signalling networks together with the remodelling of transcriptional program goes hand in hand with the reshaping of phenotypic landscapes displayed by prostate cancer cells. Mechanistic studies have not yet fully revealed the network of mutual crosstalks between particular driving events and phenotypic parameters (Figure 1). ADT represents dramatic selective pressure, which, together with the loss of REST, an AR co-repressor and master negative regulator of neurogenesis, represents an important prerequisite for the triggering of lineage plasticity [164,165,166]. N-MYC was demonstrated to be stabilized by AURKA through a kinase-independent process and, together with constitutively active AKT1 (a consequence of N-MYC-mediated AR signalling abrogation and/or PTEN inactivation) and via direct interaction with EZH2, acts as a master driver of NEPC initiated from prostate epithelial cells [122,155]. In non-small cell lung cancer (NSCLC), N-MYC has been also reported as a downstream target of SOX2 [167]. Once AR activity is intervened during ADT, the master neural transcription factor BRN2 is shown together with SOX2 to release from AR-mediated suppression and drive expression of e.g., ASCL1 and other members of pro-neural gene battery [153]. Interestingly, BRN2 and BRN4 have been both demonstrated as internal components of extracellular vesicles released by prostate cancer cells and to promote NED [154]. SOX2 itself stands as critical promoter of lineage plasticity and androgen resistance in TP53- and RB1-deficient prostate cancer; it reprograms transcriptional circuits in favour of a pro-neuronal specific gene expression pattern, which includes accumulation of ASCL1-positive neural progenitors and DCX-positive neuroblasts, both events known from SOX2-mediated adult neurogenesis in the brain [146,168,169]. Nuclear ASCL1 expression seems to persist in neuroendocrine prostate cancer cells and, moreover, DCX-positive neural progenitors from the central nervous system have been demonstrated to infiltrate prostate tumours and metastases and initiate tumour neurogenesis, thus contributing to the stabilization and promotion of neuroendocrine phenotype [106,149]. Both FOXA1 and FOXA2 factors act as pioneering chromatin remodelling factors for AR signalling and NE-specific transcription, with FOXA2 being strongly expressed in association with SYP-positive neuroendocrine prostate carcinoma samples, high-grade adenocarcinomas, and castration-resistant prostate cancer [151,152,170,171]. In mCRPC, a direct negative regulation of AR signalling (including its downstream targets such as EHF) and FOXA1 by ONECUT2 has been reported by Rotinen et al., together with increased expression of PEG10, a putative target gene of ONECUT2 being in primary PCa repressed by AR and REST [172]. ONECUT2 regulates HIF1α binding to its response elements and in synergism with SMAD3 and hypoxic conditions it activates a transcription program specific for mCRPC as well as it drives tumour aggressiveness and plasticity in NEPC [157].

Direct identification of neuroendocrine cells in mCRPC samples via their surface fingerprint may provide essential clues on how to target aggressive neuroendocrine prostate cancer cells. Interestingly, Mucin 1 (MUC1) is known as a transmembrane surface protein with an altered glycosylation pattern in prostate cancer cells [173]. Additionally, its cleavage may lead to the nuclear localization of the C-terminal part (MUC1-C), formation of chromatin-based protein interactions, and cell type context-dependent alteration of the transcription program [174]. Indeed, such a role that oncoprotein MUC1-C plays in lineage plasticity driving NEPC has been recently reported [175]. Moreover, MUC1-C is highly expressed in advanced PCa; it suppresses AR signalling, activates MYCN and BRN2 pathways, and drives expression of stemness-specific master regulators such as SOX2, NANOG, and NOTCH1 signalling [176]. Another example of surface molecule involved in NEPC development is represented by Trop2, a tumour-associated calcium signal transducer 2 (TACSTD2). Luminal epithelial cells highly positive for surface Trop2 (TACSTD2) that express high levels of SOX2 are more predisposed to NED [177]; they drive the NE phenotype together with PARP1 and are predictive of recurrence of localised PCa [178].

## 3. miRNAs as Multifaceted Crossroads Driving Neuroendocrine Prostate Cancer Development

### 3.1. miRNA Biogenesis and Mechanisms of Action

MicroRNAs (miRNAs) are evolutionarily highly conserved non-coding RNA molecules, exerting both pro- and anti-tumorigenic effects in prostate cancer [2]. Although miRNAs accomplish important regulatory functions at all stages of cancer progression, their clinical relevance in cancer diagnosis, outcome prediction, and targeted therapy is still a matter of debate and investigation [179].

More than 2000 miRNA genes in the human genome [180] are located either in protein-coding or non-coding regions of transcription units. Expression of miRNA transcripts is driven from promoter regions regulated by canonical transcription factors and epigenetic mechanisms. Several miRNAs, which are transcribed from physically adjacent miRNA genes, form a miRNA cluster [181].

miRNAs are transcribed as long hairpin molecules (pri-miRNAs) that are subsequently processed by canonical or non-canonical pathways of miRNA biogenesis. Most frequently, pri-miRNAs are cleaved to approximately 70-nt long stem-loop precursors (pre-miRNAs). Following nuclear export, the pre-miRNA is further processed into ~22 nt long mature strands, which assemble with Argonaute family proteins to form RNA-induced silencing complex (RISC). Both cleaved single strands from the 5′ and 3′ arm of the precursor miRNA can form a RISC complex in varied proportions, creating mature miRNA complexes with -5p or -3p RNA strands [182].

The RISC complex mediates downregulation of target proteins through inhibition of translation, or mRNA degradation following its deadenylation and decapping [182]. Under specific conditions, the miRNA-target interaction can induce translational activation [183]. Activation of TLR receptors by double-stranded RNA was recently identified as another mechanism of miRNA action [184].

miRNAs interact with their target mRNA molecules via imperfect base pairing. Computational miRNA-target prediction algorithms search UTRs and CDS of putative target genes for sequences complementary to the miRNA seed region, an 8-nt stretch with near-perfect base pairing. Short miRNA sequences and imperfect base-pairing imply that every miRNA can regulate a plethora of different targets and *vice versa* [185]. Only a fraction of miRNA:target interactions have been experimentally validated [186].

Mechanisms of post-transcriptional regulation of miRNAs by lncRNAs and circular endogenous RNAs are gaining increased attention for their potential to regulate miRNA and/or target availability [187,188]. Lo et al. recently discovered a novel mechanism of how the miRNA turnover can be further regulated by the interferon response pathway, through IFIT5-XRN1-mediated degradation [189].

### 3.2. miRNAs in the Regulation of NED and Prostate Cancer Progression

miRNAs can be implicated at multiple levels of NED control. The phenotypic shift towards NED can be promoted either by downregulation of miRNAs directly targeting the transcripts associated with the neuroendocrine phenotype or more frequently by modulation of expression of upstream molecules regulating the NE transformation (positive or negative). Table 2 and Supplementary Table S2 summarize the effects of selected miRNAs that were described in the context of NED phenotype or in the control of NED regulators. The purpose of these tables is to assemble current knowledge concerning miRNA expression in prostate cancer patients, potential correlation with their prognosis, validated miRNA targets relevant for the NED phenotype, and clinical utility of specific miRNA expression for cancer diagnosis or treatment prediction. Less common mechanisms of expression control are also mentioned.

As NEPC is most often associated with advanced disease, resection of tumours at this stage is rare, as it does not bring therapeutic benefit. Therefore, sources of patient samples are very limited, and very few studies actually exploit clinical samples of prostate cancer with NEPC traits for next generation sequencing analysis of transcripts. Still, findings resulting from analysis of clinical specimens can be considered biologically more relevant than observations from tissue cultures, in which the combination of artificial culture conditions and cellular plasticity can result in NE-like transformation [291]. The implication of miRNAs in the context of treatment-induced NED was recently reviewed in [292].

Bhagirath et al. have assembled a cohort of eight NEPC tumour tissues and performed sequencing of small RNAs [194], while Beltran *et al.* profiled protein-coding transcripts of seven NEPC samples by NGS [121]. miRNAs enriched [293] for the genes reported in the latter dataset, which overlapped with miRNAs deregulated in NEPC samples [194], highlighted the overexpression of miR-375 and downregulation of miR-34a, miR-30c-5p, miR-363-3p, and miR-19b-3p (Supplementary Figure S1) (detailed information about the generation of miRNAs overlay is described in Supplementary Material). Notably, increased expression of miR-375 was detected also in the serum extracellular vesicles (EVs) of patients with NEPC [217]. miR-375, miR-34a and miR-19b-3p are validated regulators of molecules associated with the NED phenotype (Table 2).

Mechanistically, miRNAs can play dual roles in prostate carcinogenesis. Opposite findings of miRNA function in biological processes implicated in cancer progression (proliferation, migration, invasion, and apoptosis) are reported for most miRNAs (Table 2 and Supplementary Table S2). These discrepancies may result from non-physiological concentrations of experimentally introduced miRNAs or miRNA antagonists, as concentrations that are typically used in transfection experiments far exceed the total cellular concentration of the most highly expressed miRNAs [188]. Most importantly, certain miRNAs such as miR-204 may act as tumour suppressors in prostate adenocarcinomas but promote cancer in neuroendocrine tumours [3].

**Figure 2:**
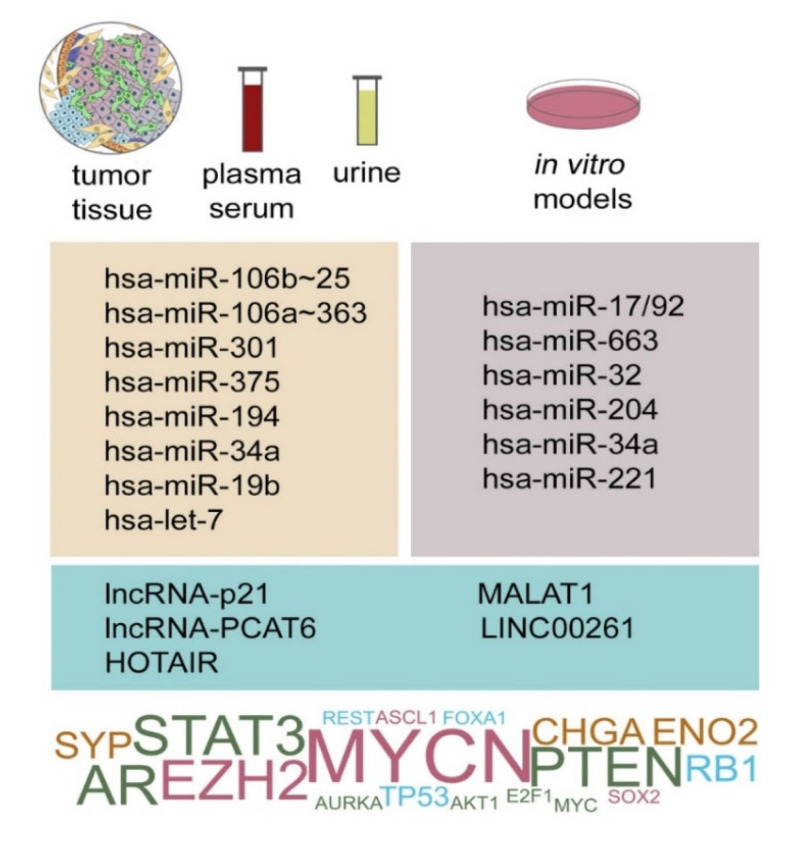
Non-coding RNAs in the regulation of NEPC. microRNAs and lncRNAs associated with the NE phenotype in clinical PCa samples (**left**) and NE-like changes in cellular models (**right**) were experimentally identified to target NED markers and regulators (bottom; dark pink, light blue, and green colours visualise driving events of NEPC based on their mode of action, i.e., upregulation, downregulation, or altered signalling status, respectively. Orange depicts NED markers. The font size reflects the frequency of a particular NED regulator/marker as a target of tested group of miRNA molecules.

The following subchapters summarize the implication of miRNAs in the NED phenotype, which were described in distinct contexts with different degrees of physiological relevance (Figure 2).

### 3.3. miRNAs Associated with Neuroendocrine Prostate Cancer

The recent availability of a next-generation sequencing methodology has enabled in-depth studies of less common phenotypes in cancer such as NEPC. Transcriptome profiling of clinical specimens identified deregulated miRNAs in prostate tumours with a neuroendocrine phenotype compared to prostate adenocarcinomas. The relevance of these findings was confirmed by validation in available patient cohorts and functional *in vitro* experiments [194]. An alternative approach exploiting Ago-HITS-CLIP based identification of miRNA binding sites revealed a correlation between miR-194 and the NE phenotype, with subsequent validation in patient samples and *in vitro* models [190]. On the other hand, the miR-106b~25 cluster and let-7 were implicated in regulatory mechanisms associated with NED induction, and their expression correlates with clinical observations [128,260]. The following information about the role of individual miRNAs and miRNA clusters associated with validated NE phenotype in PCa is summarized in Table 2.

#### 3.3.1. hsa-miR-194

miR-194 was found to be associated with plasticity of prostate cancer cells [191], and its elevated expression and activity was recently detected in NEPC [190]. Increased miR-194 inversely correlated with AR activity. Frequent gains and amplifications of miR-194, whose two copies are located on human chromosomes 1 and 11, were observed in NEPC datasets. Of the 160 putative miR-194 targets identified in the study, FOXA1 was identified as a target gene by which miR-194 influences the emergence of NEPC [190].

Upregulated miR-194 was discovered in prostatectomy specimens as well as in the circulation of relapsing patients and was associated with a higher Gleason score and poor prognosis [192,193]. By targeting SOCS2 and associated STAT3 and ERK signalling pathways, miR-194 was identified as a promoter of metastases in prostate cancer [191], although inhibition of cell motility and a negative effect on viability of cancer cells were also described [294,295].

#### 3.3.2. hsa-miR-375

miR-375 was predominantly enriched in patient tissues with NEPC features, and its experimental overexpression induced expression of NE markers SYP, ENO2, and CHGA [194]. Of NED-associated targets, hsa-miR-375 targets TP53 and NCAM1 in gastric cancer cells and neurons, respectively [195,196]. miR-375 has lately been intensively studied as a prognostic factor, and its diagnostic potential was evaluated in combination with other miRNAs elevated in advanced prostate disease [208]. In urinary exosomes, miR-375 was decreasing with disease progression [296], while in serum EVs, miR-375 expression was enriched in patients with NEPC [217]. miR-375-based non-invasive screening of circulating miRNAs could distinguish benign and aggressive disease and predict treatment response [297].

#### 3.3.3. hsa-miR-301

Similarly to miR-375, enrichment of miR-301 was detected in tumours with NEPC characteristics, and its experimental manipulation affected the NED phenotype [194]. Of NED-associated targets, miR-301 was validated to target PTEN in breast cancer [218]. Besides elevated expression in NEPC tissues, miR-301 was also increased in prostate cancer compared to adjacent tissue [220] and manifested various pro-tumorigenic effects [221,222,223,224]. In prostatectomy specimens, high levels of miR-301a were associated with higher risk of biochemical recurrence [223] and were proposed as a predictor of metastatic disease [225].

#### 3.3.4. hsa-miR-106a~363 Cluster

Six miRNAs expressed from the cluster encoded on human chromosome X (miR-106a, miR-18b, miR-19b, miR-20b, miR-92a, and miR-363) were concomitantly downregulated in NEPC [194]. Experimental downregulation of these miRNAs induced expression of NED markers CHGA, ENO2, and SYP. STAT3, MYCN, and E2F1 were identified as direct targets of the miR-106a~363 cluster [194]. miRNAs of the hsa-miR-106a~363 cluster target TP53, RB1, and PTEN tumour suppressors and negative regulators of NED [229,230,231,241,249,250,254]. In experimental settings, miR-106a~363 cluster miRNAs exhibit oncogenic properties (Table 2) and are associated with poor patient prognosis [232,244].

#### 3.3.5. hsa-miR-106b, miR-93, and miR-25 Cluster

Hsa-miR-106b was upregulated in NED arising in hypoxic conditions [260] and was significantly upregulated in multiple cohorts of patients with NEPC [121,194]. Experimentally validated targets of the miR-106b~25 cluster implicated in NED control comprise PTEN [265,268], TP53 [278], RB1 [261], and EZH2 [277]. Furthermore, the miR-106b~25 cluster downregulates the transcriptional repressor REST, which represses neuron-specific protein-coding and miRNA-coding genes. miRNAs encoded by this cluster were deregulated by hypoxia and in high grade PCa patients, and their experimental overexpression induced proneural genes in model cell lines [260].

In the rare prostatic small cell neuroendocrine carcinoma, the absence or mutation of p53 deregulated expression of miR-25 and the E3 ligase FBXW7 results in elevated levels of AURKA and enhancement of cancer cell proliferation and aggressive behaviour [276]. Amplifications of AURKA were strongly associated with the emergence of treatment-associated NED phenotype [298]. Other members of the miR-25 family, miR-92a and miR-92b, also interacted with AURKA based on NGS results, although additional evidence by a low throughput method is still lacking [299,300].

#### 3.3.6. hsa-let-7

Members of the let-7 miRNA family were implicated in the development of NEPC in the context of LIN28B signalling as its negative downstream effectors [128]. Changes in expression of multiple let-7 family members in NE cells correspond with the general function of LIN28 proteins in miRNA biogenesis [301], while the downstream regulation of AR and Myc by the LIN28B-let-7 axis influences prostate cancer progression [290]. Specifically, let-7 family members regulate multiple molecules associated with the NED phenotype: ASCL1 [284], EZH2 [281,282], MYCN [283], and HMGA-2 [128].

### 3.4. miRNAs Associated with Neuroendocrine-like Changes in Prostate Cancer Models

Experimental modulation of miRNA expression was capable of inducing an NE phenotype in cell cultures derived from prostate cancer cells, as manifested by changes in cell morphology and the induction of NE markers (Table 1). With respect to potential off-target effects of non-physiological concentrations of exogenous miRNAs [188], deregulation of miRNAs described below was frequently observed in prostate cancer, but direct association with the NE phenotype was not investigated. Detailed information about expression, function, and diagnostic utility of the following miRNAs is summarized in Supplementary Table S2.

#### 3.4.1. hsa-miR-17/92 Cluster

A well-studied miRNA cluster with oncogenic properties, encoded on human chromosome 13, encompasses six miRNAs: miR-17, miR-18a, miR-19a, miR-20a, miR-19b-1, and miR-92a-1 [302]. Upregulation of miRNAs from the miR-17/92 cluster, associated with SOX4 overexpression, was confirmed in patient sample and induced an NED phenotype in prostate cancer cells [303]. RB1 was identified as a target of miR-17/92, which is frequently absent in small cell neuroendocrine carcinoma of the prostate [304]. From other negative NED regulators, PTEN and TP53 were experimentally validated as targets of miR-17, miR-18a, miR-19a/b, miR-20a, and miR-92a [229,241,250,305,306,307,308,309,310]. On the other hand, positive regulators of NED, MYCN, and AKT1 are targeted by miR-19a and miR-19b [248,311,312].

Contrarily, downregulation of all six members of the miR-17 family, including hsa-miR-106b, was observed in experimental NED of LNCaP cells, which was associated with induction of Cyclin D1 [228]. In accordance with the Akt signalling pathway being an important regulator of NED [123], miR-17, -20b, and -106b negatively regulated AKT3, whose expression accompanied the NED phenotype in clinical samples of advanced prostate cancer [313].

#### 3.4.2. hsa-miR-663

hsa-miR-663 was characterized as an oncogenic miRNA, capable of modulating expression of the NED marker NSE in LNCaP cells. miR-663 expression correlated with Gleason score and TNM stage and was suggested as an independent prognostic predictor of clinical recurrence [314]. miR-663 was shown to target the tumour suppressor genes encoding p53 and p21 [315]. miR-663 was also identified as one of 5 miRNAs overexpressed in metastatic PCa and responsible for STAT3 deregulation [316].

#### 3.4.3. hsa-miR-32

The function of hsa-miR-32 in NED was discovered in the context of inhibited AR signalling, resulting from the interaction of prostate cancer cells with mast cells, or from anti-androgen therapy [317]. miR-32 expression was found to be regulated by AR transcriptional activity and increased in CRPC [318]. Elevated miR-32 expression was associated with pro-tumorigenic effects such as increased proliferation and transformation in vivo, chemoresistance, and radioresistance [319,320,321]. Of NED regulators, miR-32 was validated to target PTEN [322].

#### 3.4.4. hsa-miR-204 and hsa-miR-34a

A negative feedback loop, encompassing AR-driven downregulation of miR-204, inhibition or XRN-1, and downregulation of miR-34a, which enables re-expression of AR, was described in prostate cancer cells [3]. Interestingly, the described signalling loop exhibited opposite effects in PCa and NED cells, with a pro-tumorigenic effect of upregulated miR-204 on cells with the NED phenotype. In accordance with the general tumour-suppressive role of miR-34a, the expression of both miR-204 and miR-34a was found to be downregulated in advanced PCa [3,323,324,325,326]. While the expression of miR-204 is downregulated by androgens [3], miR-34a itself was validated to target AR [327] as well as other regulators of the NED phenotype MYCN [328], SOX2 [329], and TP53 [330].

#### 3.4.5. hsa-miR-221

Elevated expression of miR-221 was described in an androgen-independent subline of prostate cancer cell line LNCaP, and its experimental modulation affected the neuroendocrine phenotype of the cells, with concomitant effect on the Wnt signalling pathway through the regulation of DVL2 [331]. In prostate cancer and advanced disease, miR-221 expression was downregulated [199,332,333,334], but a significant upregulation was detected in clinical NEPC samples [194]. miR-221 was shown to affect the expression of NED regulators RB1 [335] and PTEN [336,337].

### 3.5. Additional miRNAs Implicated in the Modulation of Key Positive and Negative Regulators of NEPC

The following subchapter points out several miRNA candidates whose direct association with NED has not been experimentally demonstrated but which can regulate several positive or negative regulators of the NED phenotype: AR, MYCN, and AKT, alone or in combination with other NED-related targets.

#### 3.5.1. miRNAs Implicated in the Regulation of AR

With regard to the crucial role of AR signalling in PCa pathogenesis, a high throughput miRNA screen was performed to identify potential miRNA regulators of AR expression and transcriptional activity [338]. Besides miR-301a and miR-34a described in Section 3.3.3 and Section 3.4.4, miR-30 family members were identified as AR regulators with binding sites in both UTR and coding regions of AR; loss of miR-30c-5p and miR-30d-5p expression correlated with advanced disease [338]. Importantly, miR-30d-5p in serum EVs was deregulated in both treatment-induced and *de novo* NEPC [217]. miR-31, whose binding side in the *AR* coding region was frequently mutated in cancer, suppressed tumour formation in experimental models [339]. miR-31 negatively correlated with AR expression in a transcriptome analysis of prostate cancer tissues [340]. In general, miRNAs that were suppressed in metastatic prostate cancer, including miR-31, strongly affected AR expression and transcriptional activity and their decrease was associated with worse biochemical recurrence-free survival [341]. Notably, miR-346, miR-361-3p, and miR-197 increased AR activity through a novel and anti-dogmatic mechanism of direct association with AR 3′UTR and transcript stabilisation [342].

#### 3.5.2. miRNAs Implicated in the Regulation of AKT and MYCN

Out of 20 miRNAs experimentally validated to control the expression of AKT1 whose constitutional activation drives the emergence of the NEPC phenotype, several miRNAs were reported to be specifically implicated in prostate cancer. miR-644 was described to control several regulators of the NED phenotype in the context of CRPC, including AKT, MYC, and AR coregulators [343]. However, information about the expression or function of miR-644 in prostate cancer pathology is lacking. miR-373-3p and miR-409-3p were described as promoters of prostate tumorigenesis, migration, and invasion [344,345,346,347]. Moreover, AKT activity can be controlled indirectly such as by the mechanism involving miR-197 regulation of the VDAC1/AKT/beta-catenin pathway [348] or miR-101 control of the AKT pathway through RLIP76 [349].

AKT is a validated target of miR-27a [350], and reciprocally, upstream regulation of miR-27a expression by AR and MYC suggest a potential indirect involvement of this miRNA in NEPC [351,352,353,354]. Downregulation of miR-27a caused by aberrant AR signalling and PI3K/Akt signalling after ADT was proposed to promote the progression of castration-resistant prostate cancer [352]. Although reports of miR-27a expression in tumour samples are conflicting [352,354,355,356,357], high serum levels of miR-27a correlate with poor survival [356] and indicate presence of metastases [358]. 

#### 3.5.3. miRNAs Implicated in the Regulation of MYCN

From NED-associated miRNAs, MYCN is targeted by the above-described hsa-miR-19b, hsa-miR-let-7, and hsa-miR-34a. Hsa-miR-101 directly targets two positive regulators of NED phenotype, MYCN and EZH2 [248,359,360]. In general, miR-101 expression is often downregulated in primary and metastatic tumours [355,361,362] and, when restored, it exerts anti-cancer effects [363,364]. Its diagnostic utility for metastatic disease was proposed by several studies [253,365,366]. The expression of miR-101 can be modulated by several alternative mechanisms such as lncRNA CRNDE [367], biogenesis control by IFIT5 [189], or androgen stimulation [368].

### 3.6. LncRNAs Implicated in NEPC

Recently, next-generation sequencing data reveal novel potential RNA regulators of NED from the group of long non-coding RNAs [369]. Upregulation of lncRNA-p21 was detected in NEPC and enhanced by Enzalutamide treatment via EZH2/STAT3 signalling [370]. Differential expression of lncRNA-p21 distinguished PCa patients from BPH [371]. A similar NEPC-promoting effect was also described for lncRNA-PCAT6 by sponging miR-326 [372]. lncRNA-PCAT6 was associated with prostate cancer metastases [373]. Upregulation of LINC00261 in NEPC was discovered in patient-derived xenografts and confirmed by analysis of multiple patient cohorts [5]. Opposite trend in expression between prostate cancer and healthy tissue suggests that upregulation of LINC00261 can be specific for NEPC [374].

LncRNAs HOTAIR and MALAT1 were increased in PCa samples with neuroendocrine characteristics [340]. HOTAIR was found upregulated in CRPC and its experimental modulation regulated the NED phenotype in prostate cancer cells [375], although its function in the clinical setting was questioned [376]. Nevertheless, HOTAIR may be implicated in NED based on its ability to regulate AR degradation [377]. MALAT1 was identified as one of the most abundant transcripts in CRPC biopsies [378], and high expression of MALAT1 was proposed to stratify patients with advanced PCa who would benefit from Enzalutamide treatment [379]. Altogether, lncRNAs emerge as a novel class of RNA regulators of NEPC interacting with already known transcription factors and miRNAs, with possible diagnostic and clinical utility.

## 4. Clinical Significance of Non-Coding RNAs as Biomarkers and Therapeutic Targets in NEPC

With increasing incidence of prostate cancer, efforts to improve diagnostic and prognostic methods for patient benefit include the investigation of miRNA expression in cancer tissues and body fluids, with the scope of clinical application. The advantage of miRNAs for cancer screening is their relative abundance and stability in body fluids and straightforward quantification by PCR-based methods or alternative techniques [380]. Multiple studies detect differences in miRNA expression in biopsies or body fluids of cancer patients to stratify healthy individuals, benign prostate hyperplasia, and prostate cancer. Non-invasive collection of liquid biomarkers from blood (plasma or serum), urine, or seminal plasma can serve for prediction of disease prognosis, metastatic dissemination, and treatment outcome and to stratify patients who would benefit from therapy of advanced prostate cancer. Computational algorithms and panels of several miRNAs were designed to increase the prediction power [211,236]. Several promising candidate biomarkers of prostate cancer were proposed based on analysis of samples from circulating blood (miR-25-3p and miR-18b-5p), urine (miR-95, miR-21, miR-19a, and miR-19b), and prostatic secretions (miR-203) [381].

miRNAs can be detected both in cell-free preparations and in exosomes/extracellular vesicles (EVs), along with lncRNAs harbouring seed regions of miRNAs implicated in NEPC regulation such as let-7 family members as well as miR-17, miR-18a, miR-20a, miR-93, and miR-106b [382]. Selective excretion of miRNAs in EVs underlies the observed negative correlation between miRNA content in EVs and tumour cells. For example, the cluster comprising miR-92a was downregulated in NEPC tumours, while miR-92a iso-miRs were significantly enriched in EVs obtained from plasma of NEPC patients [154,217].

Table 2 and Supplementary Table S2 include information on possible biomarker properties of NEPC-associated miRNAs. For most miRNAs associated with the NED phenotype, a certain correlation with prostate cancer was identified, and, in most datasets, miRNAs and their combinations were characterized as better biomarkers than PSA alone. Nevertheless, only very limited information is available about miRNA implication in the diagnosis of NEPC. A recent study by Bhagirath *et al.* identified an EV-microRNA classifier in serum of six CRPC patients with NEPC features comprising miRs-9-3p, -28-5p, -378d, -592, and -155-5p [217]. These molecules can bring increased specificity to the diagnosis of NEPC along with the detection of MYCN and AURKA transcripts [298].

## 5. Conclusions and Future Perspectives

Despite many tools for experimental manipulation of miRNA expression, therapeutic application of miRNAs or miRNA antagonists remains challenging. Current clinical trials in prostate cancer involving miRNAs focus on diagnosis, disease monitoring, and prediction of treatment outcome, but therapeutic application of miRNA in advanced prostate cancer treatment is still far from implementation. Questions of drug dosage, pharmacokinetics, and delivery need to be properly addressed [383]. Besides lipidic or polymeric nanoparticle delivery of selected miRNA(s), an alternative mechanism of silencing three members of the oncogenic miR-17~92 cluster was discovered, whereby a small molecule interfering with the Dicer processing site leads to impaired miRNA biogenesis [384], and conjugation of a chemotherapeutic drug with this inhibitor represents a powerful targeting strategy of the entire oncogenic pri-miR-17-92 cluster [385].

Altogether, the current clinical utility of NEPC-associated non-coding RNAs focuses on disease diagnosis, monitoring, and prediction of treatment outcome. Nevertheless, with improved methods of delivery of miRNA-based therapeutics leading to their increased tolerability, NEPC-associated miRNAs may serve as good candidates to slow down the progression to advanced disease in prostate cancer patients.

## Figures and Tables

**Table 1:** Markers associated with NED of PCa cells, their biological function, and expression in clinical PCa and exper-imental models.

Name	Biological Function	Ref	Role in PCa/NEPC	Ref
**Chromogranin A (CgA)**	member of granin family biogenesis of secretory granules glucose and calcium homeostasis	[20,21]	NED marker elevated plasma levels associate with poor prognosis in hormone-refractory PCa IHC staining correlates with both grade and stage independent predictor of overall survival and progression-free survival in CRPC	[22,23,24,25]
**Chromogranin B (CgB)**	member of granin family secretory protein	[21]	marker of NED in prostate adenocarcinoma increased level in transdifferentiated LNCaP subclones *in vitro*	[26,27]
**Neuron-specific enolase (** **γ** **-enolase, NSE)**	isoenzyme of glycolytic enzyme enolase catalyzes conversion of 2-phosphoglycerate to phosphoenolpyruvate and its reverse reaction during gluconeogenesis expressed in cytoplasm of neurons and NE cells, erythrocytes, and platelets	[28]	increased serum level correlates with prognosis in advanced PCa, mainly mCRPC increased pretreatment NSE serum level in metastatic PCa patients correlates with poor survival elevated in mCRPC compared to clinically localized and hormone-naïve PCa	[29,30,31]
**Synaptophysin (Syn)**	membrane protein of small synaptic vesicles found also in dense-core chromaffin and neurosecretory granules incorporated in lipid bilayer forms a cation channel essential for neurosecretion	[32]	detected in metastasis of CRPC patients detected on circulating tumour cells in CRPC patients; expression correlated with resistance to enzalutamide and abiraterone acetate	[33,34]
**CD56 (N-CAM, neural cell adhesion molecule-1)**	member of immunoglobulin superfamily involved in homophilic and heterophilic interaction expression on surface of neural cells and some cells of immune system aberrant expression in haematological malignancies and solid tumours	[35]	specific NED marker in endocrine lung cancer specific surface marker of NEPC	[36,37]
**L-dopa decarboxylase (DDC)**	decarboxylation of L-Dopa to dopamine, 5-hydroxytryptophan (5-HTP) to serotonin and also other aromatic acids to corresponding amines supply organism with essential neurotransmitters implication in Parkinson’s disease	[38]	AR coactivator NED marker modulator of AR-regulated genes	[39,40,41]
**Class III β-tubulin (TUBBIII)**	tubulin formation (heterodimers with α-tubulin) constitutive expression in central and peripheral nervous system and in testes important for neural development expression induced by hypoxia and poor nutrient supply	[42]	increased after ADT in vitro expressed in CRPC patients taxane-based chemotherapy resistance	[43,44]
**Gastrin-releasing peptide (GRP)**	neuropeptide analogous to amphibian bombesin stimulation of all gastrointestinal hormones’ secretion, intestinal and pancreatic secretion, and motility exocrine and endocrine secretion, smooth muscle contraction, pain transmission mitogen, morphogen, pro-angiogenic factor in cancers	[45,46]	increased expression of GRP and receptor GRPR in response to androgen ablation in vitro GRP/GRPR signalling supports AI growth of LNCaP by increasing AR-V7 expression GRPR amplification/overexpression in CRPC GRP secretion from NE-like cells induced by GABA through GABBR1 receptor GRPR overexpression in primary PCa compared to non-neoplastic tissue (attractive target for PCa treatment)	[47,48,49]
**Calcitonin gene-related peptide (CGRP)**	result of an alternative RNA processing of the calcitonin gene vasodilator involved in cardiovascular regulation, pathophysiology of migraine, arthritis, wound healing	[50]	expressed in prostate gland in NE cells and autonomic and sensory nerves serum levels correlated with clinical stage in patients receiving hormonal therapy CGRP increases invasion of PC-3 cell line in vitro	[51,52,53]
**Proadrenomedullin N-terminal 20-peptide (PAMP)**	member of calcitonin family of peptides potent angiogenic factor	[54]	detected in CgA-positive NE cells in both normal and neoplastic prostate	[55,56]
**Adrenomedullin (AM)**	member of CGRP family produced and secreted by adrenal medulla cells, tumour cells vasodilation, cell growth, regulation of hormone secretion, apoptosis modulation, inflammatory regulation	[57]	expressed by basal cells secreted by AI cell lines in vitro production of AM by LNCaP in response to androgen withdrawal AM mediates NED in vitro and in xenografts in vivo	[55,56,58,59]
**Secretagogin**	calcium-binding protein expressed in brain, GI tract, pancreas, thyroid, adrenal medulla exocytosis, insulin synthesis and function, stress-hormone release	[60]	colocalization with CgA and NSE in both benign and cancer NE cells not stored in secretory vesicles	[61]
**Parathyroid hormone-related peptide (PTHrP)**	produced in low concentration in virtually all tissues function in transepithelial calcium transport in kidney and mammary gland, smooth muscle relaxation in uterus, bladder, GI tract, arterial wall cellular differentiation and apoptosis	[62]	increased expression in NE-transdifferentiated subclones of LNCaP in vitro protection of neighbouring PCa cells from dox-induced apoptosis stimulation of MDSC in bone marrow, which recruited to tumour tissue, stimulated PCa growth, and angiogenesis promotion of aggressive and metastatic progression of PCa through EMT induction	[27,63,64,65,66,67]
**Neurotensin (NTS)**	neurotransmitter found in CNS and GI tract paracrine or endocrine peptide in digestive and cardiovascular system growth stimulatory effect on cancer cells	[68]	induction of NTS expression in response to androgen withdrawal in LNCaP NE-transdifferentiated subclones express NTS, while parental not induction by castration in vivo NED induction in LNCaP through receptors NTSR1 and NTSR3 NTSR1 expressed in 91.8% of PCa compared to 8% of BPH NTSR1 expressed also in lymph node metastasis	[27,69,70,71,72,73]
**Vascular endothelial growth factor (VEGF)**	important factor in vasculogenesis and angiogenesis upregulation in cancers, affects tumour angiogenesis secretion by cancer cells and stroma supports endothelial cells and leads to formation of new vessels	[74]	detected in CgA-positive NE cells in PCa NEPC phenotype and angiogenesis correlation higher plasma levels in clinically localized PCa compared to healthy, and in metastatic patients compared to clinically localized preoperative plasma levels associated with biochemical progression after radical prostatectomy and LN metastasis	[75,76,77]
**Histamine**	neurotransmitter 4 types of receptors H1R/H4R	[78]	H3R overexpression in PCa vs. normal tissue, correlation with Gleason score H3R stimulates growth of LNCaP H3R expression associated with AR expression present in mast cells and in NE cells in adenomatous prostate	[78,79]
**Serotonin (5-hydroxy-tryptamine, 5-HT)**	neurotransmitter		treatment of LNCaP with 5-HT induced NED growth factor in PCa cell lines	[80,81]
**Carcinoembryonic antigen-related cell adhesion molecule 5 (CEACAM5)**	glycoprotein belonging to the family of carcinoembryonic antigen involved in adhesion and migration overexpressed in 90% of gastrointestinal, colorectal and pancreatic cancer	[82,83,84]	potential specific surface antigen of NEPC expression detected in over 60% of NEPC including patients with end-stage disease CEACAM5 antibody-drug conjugate labetuzuman govitecan showed therapeutic potential in PCa and particularly NEPC	[37,85]
**Nerve growth factor (NGF)**	member of neurotrophins regulation of growth, maintenance, and survival of certain types of neurons, control of synthesis of neuropeptides and neurotransmitters	[86]	stimulation of EMT through TrkA receptor in CRPC cell lines crosstalk between AR and NGF receptor TrkA in LNCaP increase of NGF in response to androgen deprivation promotes NED	[87,88,89]
**Neuropeptide Y (NPY)**	member of NPY family of biologically active peptides one of the most abundant neuropeptides in brain growth promoting factor in various malignancies	[90]	high expression in PCa vs. other cancers bimodal distribution in CRPC with lower levels associated with NED mCRPC regulator of nerve-PCa cells interaction, NPY-neural axis regulates apoptosis, metabolism, therapy resistance	[91,92]

**Table 2:** Cancer-related effects of miRNAs associated with NEPC.

miRNA	Associationwith NED	Validated Target			Expression in PCa Clinical Samples	Cancer-Related Effect	Prognosis Correlation with Clinical Data	Biomarker	Other Findings
		NED Marker	Positive NED Regulator	Negative NED Regulator		Experimental Findings		Source: Indication	
**hsa-miR-194**	⇗ in clinical NEPC [190]	-	-	-	⇗ in advanced disease [191]		⇗ expression in primary tumour: poor prognosis [192]	serum: BCR prediction [192] prostate biopsy: relapse prediction [193]	induces NED through FOXA1 [190]
**hsa-miR-375**	⇗ in NEPC tissues [194] ⇗ NE in cells [194]	NCAM1 [195]	-	TP53 [196]	⇗ in PCa [197,198,199] ⇗ in advanced PCa [200] ⇗ in metastatic CRPC [201]	⇗ docetaxel resistance [197] associated with epithelial phenotype [202] ⇗ proliferation, migration, tumour growth [203] dual effect on malignant phenotype [200]	poor overall survival [204] relapse after radiotherapy, shorter overall survival [205] early progression [201] association with baseline CTC count and PSA response [206]	serum: PCa vs healthy [122,207] serum: BPH vs PCa [208] urine: BPH vs PCa [209] urine: disseminated vs localized [210] plasma: treatment outcome prediction [204,205] plasma: disease staging [211] plasma: metastasis prediction [212,213] serum: advanced disease [214]	correlates with CTCs in metastatic patients [202] positive correlation with AR expression [215] enriched in epithelial cells [216] ⇗ in NEPC patient datasets [121,194,217]
**hsa-miR-301a**	enriched in NEPC tissues [194] induces NE in cells [194]	-	-	PTEN [218] AR [219]	⇗ in prostate tumour relative to adjacent tissue [220]	⇗ proliferation [221] ⇗ radioresistance [222] ⇗ EMT [223] ⇗ migration, invasion [224]	increased risk of BCR [223] high predicts metastasis [225]	serum, tumour: BPH vs PCa [226] serum, needle biopsy: low grade tumours [227]	⇗ by hypoxia [222] ⇗ by hyperglycemia [221]
**hsa-miR-106a**	⇘ in NEPC tissues [194] ⇘ in experimental NED [228]	-	-	TP53 [229] PTEN [230] RB1 [231]	⇗ in high grade tumours [232] ⇘ expression with ⇗ malignity [233] ⇗ in solid tumours [234]	⇗ proliferation and metastasis [235] confers radioresistance [232]	⇗ expression - BCR [236]	blood: ⇗ predicts BCR [236] serum: localized PCa vs BPH [237] serum: low risk vs aggressive PCa [238]	regulated by lncRNAs HAND2-AS1 [239] FER1LR [240]
**hsa-miR-92a**	-	-	-	PTEN [241] TP53 [229]	⇘ [242,243] ⇗ in solid tumours of different origin [234]	⇘ viability, migration, invasion [242] ⇗ viability, migration, invasion [244] ⇗ proliferation [245]	-	urine: PCa vs BPH vs healthy [246]	regulated by lncRNA FER1LR [247]
**hsa-miR-19b**	⇘ in NEPC tissues [194]	-	MYCN [248]	PTEN [249] TP53 [250]	-	⇗ proliferation [245]	-	plasma: localized vs metastatic PCa [251] urine: BCR [252] urine, urine EVs, plasma: PCa vs BPH vs healthy [246] biopsy: tumour vs adjacent tissue [253]	-
**hsa-miR-20b**	⇘ in NED *in vitro* [228]	-	-	PTEN [254]	⇗ in PCa vs adjacent tissue [244,255]	⇘ migration, invasion, EMT [256] ⇗ proliferation, migration [255]	poor survival [244]	tissue: ⇗ predicts BCR [236]	regulated by lncRNA PART1 [257]
**hsa-miR-363**	⇘ in NEPC tissues [194]	-	-	-	⇗ in recurrent PCa [258] ⇘ in young PCa patients [199] ⇘ in expression concomitantly with an ⇗ in malignancy [233]	⇗ proliferation and EMT [259]	-	-	miR-363 biogenesis regulated by IFIT5, downstream of IFNgamma - antiviral response) [189]
**hsa-miR-106b**	⇗ in NED from hypoxia [260] ⇘ in experimental NED [228]	-	-	RB1 [261] PTEN [262] TP53 [229]	⇗ in PCa vs BPH [263] ⇗ in PCa [264] [265] ⇗ in PCa and metastases [266]	⇗ viability, migration, invasion [264] overrides radiation-induced cell cycle arrest [267]	associated with disease recurrence [266]	-	-
**hsa-miR-93**	⇘ in experimental NED [228]	-	-	PTEN [268]	⇗ in PCa [199] ⇗ in patients with LN metastases [269]	promotes PCa progression [270,271]	⇗ expression predicts poor survival [272]	blood: BPH vs PCa [208] seminal plasma: disease aggressiveness [273] plasma: disease prediction [274] serum: PCa diagnosis [275]	-
**hsa-miR-25**	⇗ in NED from hypoxia [260] ⇗ in small cell neuroendocrine carcinoma [276]	-	EZH2 [277]	TP53 [278] PTEN [265]	⇗ in PCa [199] ⇗ in advanced PCa [260] ⇗ in patients with LN metastases [269]	⇘ invasiveness [279]	-	serum: disease stage and risk [233] serum: decreased in cancer [280]	-
**hsa-let-7**	⇘ in NEPC [128]	-	EZH2 [281,282] HMGA2/SOX2 [128] MYCN [283] ASCL1 [284]	-	⇘ in advanced PCa [285]	⇘ favors progression and self-renewal [129]	⇘ correlates with early clinical failure [286]	urine: cancer cell - macrophage signalling [287] urine: PCa vs healthy [288]	negative regulation by lncRNA TTTY15 [289] suppresses AR via Myc [290]

## Data Availability

Openly available datasets have been analyzed and cited in this study—Supplementary Table S3 in [121] at 10.1158/2159-8290.CD-11-0130. Supplementary Table S2 in [194] at/10.1038/s41388-020-01493-8.

## Supplementary Materials

The following are available online at https://www.mdpi.com/article/10.3390/ncrna7040075/s1, Figure S1: Candidate miRNAs involved in prostate cancer neuroendocrine differentiation, Table S1: Regulators and markers of neuroendocrine differentiation in prostate cancer, Table S2: Cancer-related effects of NED-associated non-coding RNAs. References [386,387,388,389,390,391,392,393,394,395,396,397,398,399,400,401,402,403,404,405,406,407,408,409,410,411,412,413,414,415,416,417,418,419,420,421,422,423,424,425,426,427,428,429,430,431,432,433,434,435,436,437,438,439,440,441,442,443,444,445,446,447,448,449,450,451,452,453,454,455,456,457,458,459,460,461,462,463,464,465,466,467,468,469,470,471,472,473,474,475,476,477,478,479,480,481,482,483] are refered to in Supplementary Materials.

## Abbreviations

ADT	androgen-deprivation therapy
Ago-HITS-CLIP	high-throughput sequencing of RNA isolated by cross-linking immunoprecipitation of Argonaute protein
AI	androgen independence
AKT1 AR	AKT Serine/threonine Kinase 1 androgen receptor
ARPIs ASCL1 AURKA	AR pathway inhibitors Achaete-Scute Family BHLH Transcription Factor 1 Aurora kinase A
BCR	biochemical recurrence
BPH BRN2 BRN4 CgA (*CHGA*)	benign prostatic hyperplasia POU Class 3 Homeobox 2 POU Class 3 Homeobox 4 chromogranin A
CRPC	castration-resistant prostate cancer
CTC	circulating tumour cell
DCX E2F1 EHF EMT	Doublecortin E2F Transcription Factor 1 ETS Homologous Factor epithelial-to-mesenchymal transition
EVs	extracellular vesicles
EZH2 FOXA1 FOXA2 HOTAIR HRPCa	Enhancer Of Zeste 2 Polycomb Repressive Complex 2 Subunit Forkhead box A1 Forkhead box A2 HOX transcript antisense RNA hormone refractory prostate cancer
LINC00261 lncRNA	Long Intergenic Non-Protein Coding RNA 261 long non-coding RNA
lncRNA-PCAT6 m-CRPC	long non-coding RNA prostate cancer-associated transcript 6 metastatic castration-resistant prostate cancer
MALAT1 miRNA	Metastasis Associated Lung Adenocarcinoma Transcript 1 microRNA
MUC1-C N-MYC (*MYCN*) NE cells	Mucin 1 C-terminal part Neuroblastoma MYC oncogene neuroendocrine cells
NE-like cancer cells	neuroendocrine-like cancer cells
NED	neuroendocrine differentiation
NEPC	neuroendocrine prostate cancer
NSE (*ENO2*)	neuron-specific enolase
ONECUT2 PARP1 PCa	One cut homeobox 2 Poly (ADP-ribose) polymerase 1 prostate cancer
PEG10 PSA	Paternally Expressed 10 prostate specific antigen
PTEN RB1 REST SCC	Phosphatase and tensin homolog RB Transcriptional Corepressor 1 RE1 Silencing Transcription Factor small cell carcinoma
SOX2 SRRM4 STAT3 Syp	Sex-determining region Y2 Serine/Arginine Repetitive Matrix 4 Signal Transducer And Activator Of Transcription 3 synaptophysin
t-NEPC TF Trop-2	treatment-emergent neuroendocrine prostate cancer transcription factor Tumor Associated Calcium Signal Transducer 2
