# Supplementary material for: Regulation of Neuroendocrine-like Differentiation in Prostate Cancer by Non-Coding RNAs
Source: Noncoding RNA. 2021 Dec 2;7(4):75. doi: 10.3390/ncrna7040075 (PMC8704250; doi:10.3390/ncrna7040075)
Supplement: Supplementary file 1 [file ncrna-07-00075-s001.zip › ncrna-1391656-supplementary.pdf]

non-coding RNA

MDPI

Supplementary Materials

Regulation of Neuroendocrine-like Differentiation in Prostate Cancer by Non-Coding RNAs

Eva Slabáková †, Zuzana Kahounová †, Jiřina Procházková † and Karel Souček *

Department of Cytokinetics, Institute of Biophysics of the Czech Academy of Sciences, 61265 Brno, Czech Republic; slabakova@ibp.cz (E.S.); pernicova@ibp.cz (Z.K.); prochazkova@ibp.cz (J.P.)
* Correspondence: ksoucek@ibp.cz; Tel.: +420-541-517-166
† Authors contributed equally to this study.

Supplementary Methods

S1.1 Validated miRNAs targeting regulators and markers of neuroendocrine differentiation. Based on extensive literature mining, we identified genes encoding regulators and markers involved in neuroendocrine differentiation of prostate cancer (Supplementary Table 1). These genes were used as inputs for miRTarBase [1] in order to reveal validated miRNAs targeting their expression (in Supplementary Figure 1 assigned as "validated miRNAs").

S1.2 miRNA enrichment analysis of genes differentially expressed in samples of neuroendocrine prostate cancer vs. prostate adenocarcinoma. Using the Toppfun tool [2], we performed miRNA enrichment analysis of upregulated and downregulated genes identified in the RNA-seq study published by Beltran et al. [3] that were differentially expressed in the clinical samples of primary prostate adenocarcinoma and neuroendocrine prostate cancer. Genes with logFC values > 1.5 or < -1.5 and adjusted P value < 0.05 were used as an input geneset for the Toppfun tool. miRNA hits with Bonferroni p value < 0.05 were considered significant and were used for the identification of overlay between tested datasets (in Supplementary Figure 1 assigned as "enriched miRNAs").

Published dataset analyzed in this article

[3] Supplementary Table S3 in original manuscript—GOIs differentially expressed in NEPC vs. PCA clinical samples

[4] Supplementary Table S2 in original manuscript—miRNA genes differentially expressed in CRPC-NE vs. CRPC-Adeno clinical samples

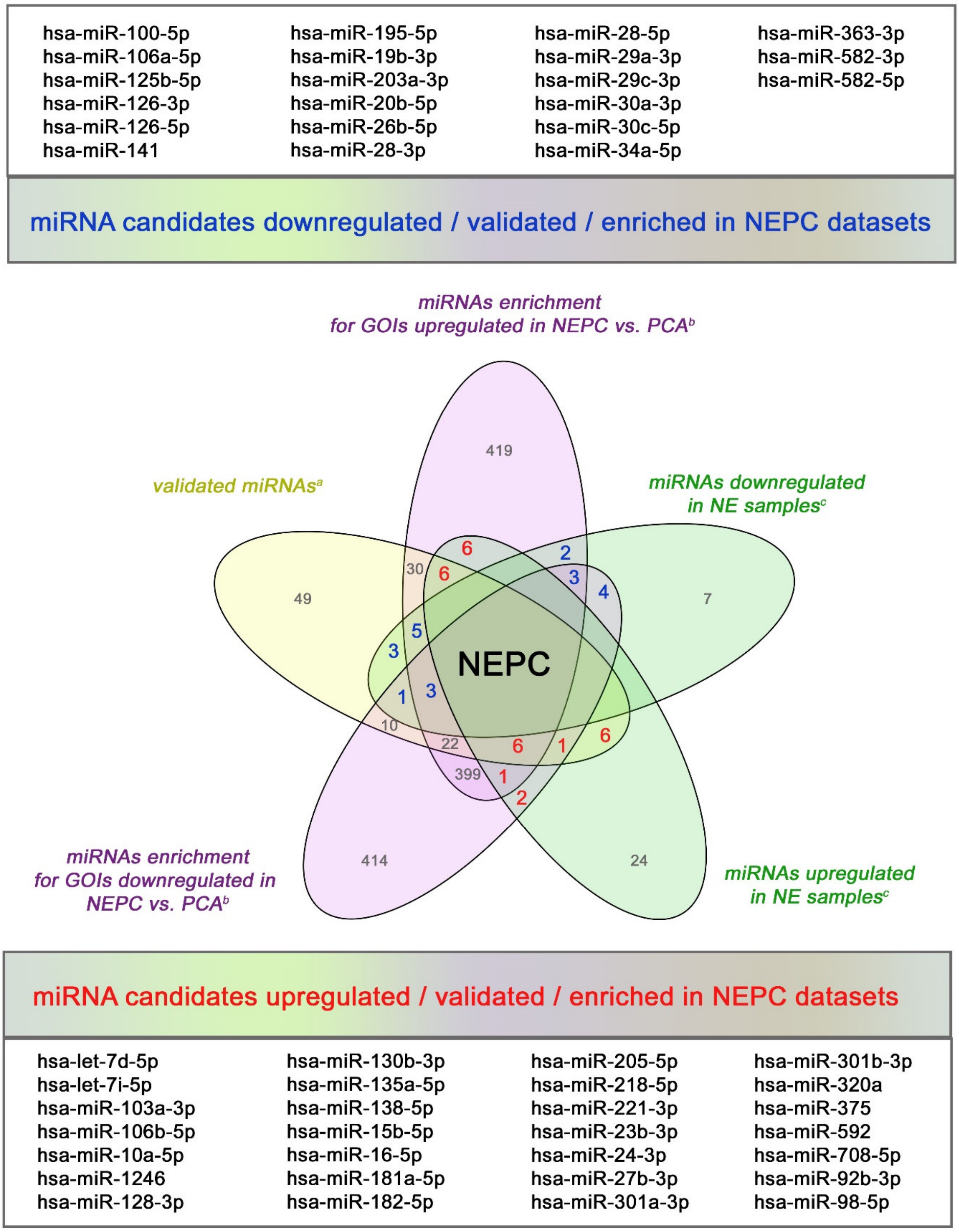

Supplementary Figure S1. Candidate miRNAs involved in prostate cancer neuroendocrine differentiation**.** miRNA candidates related to the neuroendocrine prostate cancer (NEPC) development were identified across three distinct datasets. The first dataset of miRNA candidates was created through identification of miRNAs previously validated to target regulators and markers of NEPC by using miRTarBase [1]. Supplementary methods—S1.1; in Venn diagram geneset assigned by letter 'a'). Second dataset of miRNA candidates was derived from genes of interest (GOIs) previously identified by RNA-seq analysis comparing expression of genes differentially expressed between primary prostate adenocarcinoma (PCA) and neuroendocrine prostate carcinoma (NEPC) [3] (Supplementary methods—S1.2; in Venn diagram genesets assigned by letter 'b'). The third dataset of miRNA candidates originates from the study by Bhagirath et al., where genes encoding miRNAs differentially expressed between CRPC-NE vs. CRPC-Adeno clinical samples were reported [4] (in Venn diagram genesets assigned by the letter 'c'). miRNA candidates that were upregulated in NEPC-

related datasets [4], experimentally validated to target selected regulators and markers of NEPC (our investigation) and, simultaneously, calculated as significantly enriched for geneset differentially expressed in NEPC vs. PCA samples [3], are summarized in lower table (in total 28; visualised in red). Similarly, miRNA candidates downregulated in NEPC-related datasets [4], experimentally validated to target selected regulators and markers of NEPC and, simultaneously calculated as significantly enriched for geneset differentially expressed in NEPC vs. PCA, are summarized in upper table (in total, 21; visualised in blue).

Supplementary Table S1. Regulators and markers of neuroendocrine differentiation in prostate cancer. Table shows selected genes chosen as targets for identification of experimentally validated miRNAs (miRTarBase; Supplementary methods—S1.1).

	**Regulators and markers of NEPC**
**Abbreviation / Synonym**	**Description**
hASH1/ ASCL1/ Mash1	Achaete-Scute Family BHLH Transcription Factor 1
AKT1	AKT Serine/Threonine Kinase 1
AR	Androgen receptor
BRN2 / POU3F2	POU Class 3 Homeobox 2
DCX	Doublecortin
DDC	Dopa Decarboxylase
EZH2	Enhancer Of Zeste 2 Polycomb Repressive Complex 2 Subunit
FOXA1	Forkhead Box A1
FOXA2	Forkhead Box A2
N-MYC / MYCN	Neuroblastoma MYC oncogene
NCAM1 / CD56	Neural Cell Adhesion Molecule 1
ONECUT2/ HNF6-beta	One cut homeobox 2
PTEN	Phosphatase and tensin homolog
PTHLH	Parathyroid Hormone Like Hormone
RB1	RB Transcriptional Corepressor 1
REST	RE1 Silencing Transcription Factor
SNAP25	Synaptosome Associated Protein 25
SOX2	Sex-determining region Y 2
Trop2 / TACSTD2	Tumor Associated Calcium Signal Transducer 2
TP53	Tumor Protein P53
TUBB3	Tubulin Beta 3 Class III

Supplementary Table 2. Cancer-related effects of NED-associated non-coding RNAs.

	Association with NED	Validated target			Expression in PCa	Cancer-related effect	Prognosis	Biomarker	Other findings
		NED marker	positive NED regulator	negative NED regulator	clinical samples	experimental findings	correlation with clinical data	source: indication	
**1. miRNAs associated with neuroendocrine-like changes in prostate cancer models**									
hsa-miR-17~92 cluster						• ⬂ proliferation, migration, tumour growth, enhanced sensitivity to anti-androgens [5]<br>• conveys chemoresistance [6]	• ⬀ -> higher Gleason score, shorter BCR [7]<br>• ⬀ -> poor prognosis [8]	-	• seed sequence enriched in exosomal lncRNAs from PCa cells [9]
**hsa-miR-17**	• ⬂ in experimental NED [10]<br>• ⬂ in NE-transformed LNCaP [11]	-	-	• RB1 [12]<br>• PTEN [13]<br>• TP53 [14]	• ⬀ in solid tumours of different origin [15]<br>• ⬂ in high grade tumours [16]<br>• associated with metastases [17]	• ⬂ proliferation, ⬀ apoptosis [18]<br>• ⬀ radiosensitivity [19]<br>• ⬀ proliferation, invasion, tumour growth [20]<br>• modulates androgen signalling [21]	• extreme high and extreme low expression in aggressive disease [22]<br>• high expression -> BCR [23]	• serum: PCa vs BPH [24]	• regulated by circITCH [25] and circ-MTO1 [26]<br>• regulates mitochondrial antioxidant enzymes [27]

	Association with NED	Validated target			Expression in PCa	Cancer-related effect	Prognosis	Biomarker	Other findings
		NED marker	positive NED regulator	negative NED regulator	clinical samples	experimental findings	correlation with clinical data	source: indication	
**hsa-miR-18a**	-	-	-	• PTEN [28]	-	• ↗ proliferation [29]<br>• ↗ tumorigenesis [30]	-	• plasma: healthy vs PCa [31]<br>• blood: ↗ in advanced disease [32]	• regulated by lncRNAs GAS5 [33],<br>• FENDRR [34],<br>• PART1 [35]
**hsa-miR-19a**	• ↗ in the context of SOX4 upregulation [8]	-	-	-	-	• ↗ tumorigenesis [36]<br>• prevents metastasis [37]<br>• ↗ PCa progression [38]<br>• regulates proliferation in CRPC [39]	-	• serum: predict adverse pathology [40]<br>• urine: BCR prediction [41]	• ↗ by AR [42]
**hsa-miR-20a**	• ↘ in experimental NED [10]<br>• ↗ in the context of SOX4 upregulation [8]<br>• ↘ in NE-transformed LNCaP [11]	-	-	• RB1 [12]<br>• TP53 [14]	• ↗ in solid tumours of different origin [15]<br>• ↗ in advanced tumours [43–45]<br>• ↗ in cancer vs healthy tissue [46]	• ↗migration, invasion [43]<br>• ↗ tumour growth [47]<br>• prevents apoptosis [48]	• change after docetaxel predicts survival [49]	• blood, plasma, serum: PCa vs non-cancer [45,50,51]	-
**hsa-miR-19b**	• ↘ in NEPC tissues [4]					-	• MYCN	• PTEN	

	Association with NED	Validated target			Expression in PCa	Cancer-related effect	Prognosis	Biomarker	Other findings
		NED marker	positive NED regulator	negative NED regulator	clinical samples	experimental findings	correlation with clinical data	source: indication	
							[52]	[53]<br>• TP53 [54]	
**hsa-miR-92a**	-				-	-	• PTEN [55]<br>• TP53 [14]	• ↘ [56,57]<br>↗ in solid tumours of different origin [15]	• ↘ viability, migration, invasion [56]<br>• ↗ viability, migration, invasion [58]<br>↗ proliferation [59]
**hsa-miR-663**	• ↗ NED *in vitro* [60]	-	-	• TP53 [61]	-	-	• ↗ expression → clinical recurrence [60]	-	-
**hsa-miR-32**	• mediates enzalutamide-induced NED [62]	-	-	• PTEN [63]	• ↘ in blood [51]<br>• enriched in exosomes from chemoresistant cells [64]	• ↗ cisplatin resistance [65]<br>• ↗ proliferation and promotes transformation [66]<br>• ↗ radioresistance [67]	-	• metaanalysis localized vs metastatic [68]<br>• tissue: BPH vs CaP [69,70]	• regulated by AR [71]

	**Association with NED**	**Validated target**			**Expression in PCa**	**Cancer-related effect**	**Prognosis**	**Biomarker**	**Other findings**
		**NED marker**	**positive NED regulator**	**negative NED regulator**	**clinical samples**	**experimental findings**	**correlation with clinical data**	**source: indication**	
**hsa-miR-204**	• ⬀ with NED with tumour promoting effect [72]	-	-	-	• ⬂ in PCa [72]<br>• ⬂ in advanced PCa [73]<br>• ⬂ in tumours compared to adjacent tissue [74]<br>• ⬀ in blood, ⬂ in cancer tissue [51]	• ⬂ migration, invasion, bone metastases [73]<br>• ⬀ docetaxel sensitivity [75]<br>• ⬀ apoptosis [76]	• ⬂ expression → poor prognosis [73]	• metaanalysis: PCa vs BPH [68]<br>• urine: BCR prediction [77]<br>• urine: isomiRs in healthy vs PCa [78]<br>• tumour: primary vs metastatic [79]	• expression negatively regulated by androgens [72]<br>• negative regulation by lncRNAs NEAT1 [80], UCA1 [81,82]<br>• ⬀ AR, prevents TMPRSS2/ERG fusion [83]<br>• ⬀ in exosomes in hypoxia [84]
**hsa-miR-34a**	• downstream effector in the AR-miR-204-XRN1 axis with tumour promoting effect on NED cells [72]	-	• MYCN [85]<br>• SOX2 [86]	• AR [87]<br>• TP53 [88]	• ⬂ in advanced tumours [89,90]	• ⬀ docetaxel sensitivity [91]<br>• ⬂ IL-6 induced EMT and invasion [92]<br>• ⬂ proliferation [93]	• ⬂ expression associated with poor prognosis [96]	• EVs *in vitro*: docetaxel resistance [97]<br>• Metaanalysis: PCa vs BPH [68]	• negative regulation by lncRNAs NEAT1 [80], DANCR [98], LINC01006 and LINC00662

	Association with NED	Validated target			Expression in PCa	Cancer-related effect	Prognosis	Biomarker	Other findings
		NED marker	positive NED regulator	negative NED regulator	clinical samples	experimental findings	correlation with clinical data	source: indication	
						• ⬂ migration and invasion [94]<br>• epigenetic silencing induces chemoresistance [95]			[99,100]<br>• targets AR [87]<br>• Frequent silencing by CpG methylation [101]
**hsa-miR-221**	• OE ⇨ NED of LNCaP [102]	-	-	• PTEN [103]<br>• RB1 [104]	• ⬂ in PCa [105] [106,107]<br>• ⬂ in advanced disease [108]	• sensitize to TRAIL [109]<br>• ⬂ proliferation, migration, metastasis [105]	• associated with BCR [110]<br>• ⬂ in recurrent PCa [111]<br>• ⬂ expression associated with worse BCR free survival [112]	• plasma: ⬀ in PCa, less in advanced [102]<br>• blood: localized vs metastatic [31]	-
**2. miRNAs implicated in modulation of positive and negative regulators of NEPC**									
**hsa-miR-101**	-	-	• MYCN [52]	-	• ⬂ in PCa [115]<br>• ⬂ in metastatic PCa	• ⬂ prostate cancer cell growth [118]	-	• tissue: metastatic patients [120]<br>• serum: ⬂ in PCa [121]	• regulated by lncRNA CRNDE [123]

	Association with NED	Validated target			Expression in PCa	Cancer-related effect	Prognosis	Biomarker	Other findings
		NED marker	positive NED regulator	negative NED regulator	clinical samples	experimental findings	correlation with clinical data	source: indication	
			• EZH2 [113,114]		[116,117]	• ⬂ migration, invasion, tumour growth [119]		• putative biomarker of PCa metastasis [79,122]	• miR-101 biogenesis regulated by IFIT5 (downstream of IFNγ -antiviral response) [124] • expression modulated by androgen stimulation [125]
**hsa-miR-27a**	-	-	• AKT [126]	• TP53 [127]	• ⬂ in metastatic PCa [116] • ⬀ in PCa [128] • ⬂ in PCa [129,130] • enriched in EVs of patients with advanced disease [131]	• contributes to chemoresistance [132]	• ⬀ serum levels correlate with poor survival [128] • associated with metastases [17]	• serum: BPH vs CaP [133]	• regulated by androgen [42,129,134] • regulated by MYC [130] • regulated by lncRNAs PVT-1 [135] and ZFAS1 [136]
**hsa-miR-30c-5p**	-	-	-	• TP53 [137]	• ⬂ in PCa and CRPC [138]	-	-	• urine: BPH vs PCa [140]	-

	Association with NED	Validated target			Expression in PCa	Cancer-related effect	Prognosis	Biomarker	Other findings
		NED marker	positive NED regulator	negative NED regulator	clinical samples	experimental findings	correlation with clinical data	source: indication	
					• ↘ in PCa, associated with metastatic disease [139]			• urine: prediction of biopsy results [77]<br>• tissue: BCR prognosis [141]	
**hsa-miR-30c-1-3p**	-	-	-	-	• ↘ in PCa [142]	• ↘ proliferation by restricting AR-V7 expression [142]	-	-	-
**hsa-miR-30d-5p**	-	-	• EZH2 [143]	• TP53 [144]	• ↘ in PCa and CRPC [138]	• ↘ cancer cell growth and invasion [145]	-	• seminal plasma: prediction of disease aggressiveness [146]	-
**hsa-miR-31**	-	-	-	-	• ↘ in cancer tissue [147]<br>• ↘ in cancer, increasing in high grade [148]<br>• ↘ in primary cancer, increased in metastatic [149]	• ↘ proliferation and invasion, induces apoptosis [150]<br>• ↘ tumour growth *in vivo* [151]<br>• restores sensitivity to chemotherapy [152]	• promoter methylation promotes BCR free survival [153]<br>• ↘ expression associated with worse BCR free survival [112]	• extracellular vesicles: healthy vs BPH [154]	-
**hsa-miR-346**	-	-	-	-	-	• ↗ AR activity through a novel and anti-dogmatic mechanism of direct association with AR 6.9	-	-	-
**hsa-miR-361-3p**	-	-	-	-	• ↘ in recurrent cancer [156]<br>• ↗ in recurrent cancer [111]		-	-	-

	Association with NED	Validated target			Expression in PCa	Cancer-related effect	Prognosis	Biomarker	Other findings
		NED marker	positive NED regulator	negative NED regulator	clinical samples	experimental findings	correlation with clinical data	source: indication	
					• ↘ in CRPC [157]	kb 3'UTR and transcript stabilization [155]			
**hsa-miR-197**		-	-	-	• ↗ in CRPC cell lines [158] • ↗ in PCa tissues [159]		-	-	-
**hsa-miR-644**	-	-	• AKT [160]	-	-	-	-	-	-
**hsa- miR-373-3p**	-	-	• AKT [161]	-	• ↗ in high grade PCa [162]	• regulates invasion by TGF-β signalling [163] • ↗ migration and invasion [164]	-	-	-
**hsa- miR-409-3p**	-	-	• AKT [165]	-	-	• ↗ tumorigenesis, EMT, metastasis [166,167]	-	• plasma: CaP diagnosis [168] • serum: low risk vs CRPC [169] • serum exosomes: prediction of radiotherapy benefit [170]	

	Association with NED	Validated target			Expression in PCa	Cancer-related effect	Prognosis	Biomarker	Other findings
		NED marker	positive NED regulator	negative NED regulator	clinical samples	experimental findings	correlation with clinical data	source: indication	
**3. lncRNAs associated with neuroendocrine-like changes in prostate cancer models**									
**lncRNA-p21**	• ⬀ NSE, CgA, SYP [171]	• CgA, NSE, SYP [171]	• EZH2 [171]	-	• ⬀ in NEPC [171]	-	-	• urine: distinguish BPH from PCa [172]	• expression controlled by AR [171] • competes with lncRNA-HOTAIR in EZH2 binding [171]
**lncRNA-PCAT6**	• ⬀ NSE, CgA, and SYP [173]	-	-	-	• ⬀ expressed in NEPC [173]	-	• ⬀ expression predicts poor prognosis [174]	-	• ⬀ tumorigenesis by sponging miR-326 [173]
**HOTAIR**	• ⬀ in NE-like tumours [175] • positive correlation with CHGA [175]	-	• EZH2 [176]	-	• ⬀ in NED and CRPC [177]	• ⬀ migration and invasion by regulating hepaCAM [178] • ⬀ proliferation and invasion [179] • ⬀ stem-like phenotype and docetaxel resistance [180]	-	-	• regulated by REST [177] • ⬂ miR-193a [181] • regulated by androgens, stabilizes AR [179]
**MALAT1**	• ⬀ in NE-like tumours [175]	-	• cofactor of EZH2 [182]	-	• ⬀ expressed in PCa tumours [183] • ⬀ in PCa [184]	• ⬀ docetaxel resistance [185]	• ⬀ expression associated with poor survival [183]	• urine, plasma: PCa diagnosis [187–189]	• regulates miR-1 [190] • regulates miR-320 [190]

	**Association with NED**	**Validated target**			**Expression in PCa**	**Cancer-related effect**	**Prognosis**	**Biomarker**	**Other findings**
		**NED marker**	**positive NED regulator**	**negative NED regulator**	**clinical samples**	**experimental findings**	**correlation with clinical data**	**source: indication**	
							• enzalutamide treatment prediction [186]		• regulates miR-145-5p [185]
**LINC00261**	• ⬀ in NEPC [191]	-	-	-	• ⬂ in PCa [192]	• ⬀ tumour formation and invasion [191]<br>• ⬀ by radiotherapy [193]	-	-	• drives NEPC through miR-8485-CBX2-FOXA2 [191]

References

1. Huang, H.Y.; Lin, Y.C.; Li, J.; Huang, K.Y.; Shrestha, S.; Hong, H.C.; Tang, Y.; Chen, Y.G.; Jin, C.N.; Yu, Y.; et al. miRTarBase 2020: updates to the experimentally validated microRNA-target interaction database. *Nucleic Acids Res* **2020**, *48*, D148-D154, doi:10.1093/nar/gkz896.
2. Chen, J.; Bardes, E.E.; Aronow, B.J.; Jegga, A.G. ToppGene Suite for gene list enrichment analysis and candidate gene prioritization. *Nucleic Acids Res* **2009**, *37*, W305-311, doi:10.1093/nar/gkp427.
3. Beltran, H.; Rickman, D.S.; Park, K.; Chae, S.S.; Sboner, A.; MacDonald, T.Y.; Wang, Y.; Sheikh, K.L.; Terry, S.; Tagawa, S.T.; et al. Molecular characterization of neuroendocrine prostate cancer and identification of new drug targets. *Cancer Discov* **2011**, *1*, 487-495, doi:10.1158/2159-8290.CD-11-0130.
4. Bhagirath, D.; Liston, M.; Patel, N.; Akoto, T.; Lui, B.; Yang, T.L.; To, D.M.; Majid, S.; Dahiya, R.; Tabatabai, Z.L.; et al. MicroRNA determinants of neuroendocrine differentiation in metastatic castration-resistant prostate cancer. *Oncogene* **2020**, *39*, 7209-7223, doi:10.1038/s41388-020-01493-8.
5. Ottman, R.; Levy, J.; Grizzle, W.E.; Chakrabarti, R. The other face of miR-17-92a cluster, exhibiting tumor suppressor effects in prostate cancer. *Oncotarget* **2016**, *7*, 73739-73753, doi:10.18632/oncotarget.12061.
6. Zhou, P.; Ma, L.; Zhou, J.; Jiang, M.; Rao, E.; Zhao, Y.; Guo, F. miR-17-92 plays an oncogenic role and conveys chemo-resistance to cisplatin in human prostate cancer cells. *Int J Oncol* **2016**, *48*, 1737-1748, doi:10.3892/ijo.2016.3392.
7. Feng, S.; Qian, X.; Li, H.; Zhang, X. Combinations of elevated tissue miRNA-17-92 cluster expression and serum prostate-specific antigen as potential diagnostic biomarkers for prostate cancer. *Oncol Lett* **2017**, *14*, 6943-6949, doi:10.3892/ol.2017.7026.
8. Liu, H.; Wu, Z.; Zhou, H.; Cai, W.; Li, X.; Hu, J.; Gao, L.; Feng, T.; Wang, L.; Peng, X.; et al. The SOX4/miR-17-92/RB1 Axis Promotes Prostate Cancer Progression. *Neoplasia* **2019**, *21*, 765-776, doi:10.1016/j.neo.2019.05.007.
9. Ahadi, A.; Brennan, S.; Kennedy, P.J.; Hutvagner, G.; Tran, N. Long non-coding RNAs harboring miRNA seed regions are enriched in prostate cancer exosomes. *Sci Rep* **2016**, *6*, 24922, doi:10.1038/srep24922.
10. Dankert, J.T.; Wiesehofer, M.; Czyrnik, E.D.; Singer, B.B.; von Ostau, N.; Wennemuth, G. The deregulation of miR-17/CCND1 axis during neuroendocrine transdifferentiation of LNCaP prostate cancer cells. *PLoS One* **2018**, *13*, e0200472, doi:10.1371/journal.pone.0200472.
11. Wiesehofer, M.; Czyrnik, E.D.; Spahn, M.; Ting, S.; Reis, H.; Dankert, J.T.; Wennemuth, G. Increased Expression of AKT3 in Neuroendocrine Differentiated Prostate Cancer Cells Alters the Response Towards Anti-Androgen Treatment. *Cancers (Basel)* **2021**, *13*, doi:10.3390/cancers13030578.
12. Trompeter, H.I.; Abbad, H.; Iwaniuk, K.M.; Hafner, M.; Renwick, N.; Tuschl, T.; Schira, J.; Muller, H.W.; Wernet, P. MicroRNAs MiR-17, MiR-20a, and MiR-106b act in concert to modulate E2F activity on cell cycle arrest during neuronal lineage differentiation of USSC. *PLoS One* **2011**, *6*, e16138, doi:10.1371/journal.pone.0016138.
13. Hu, H.; Li, H.; He, Y. MicroRNA-17 downregulates expression of the PTEN gene to promote the occurrence and development of adenomyosis. *Exp Ther Med* **2017**, *14*, 3805-3811, doi:10.3892/etm.2017.5013.
14. Arabi, L.; Gsponer, J.R.; Smida, J.; Nathrath, M.; Perrina, V.; Jundt, G.; Ruiz, C.; Quagliata, L.; Baumhoer, D. Upregulation of the miR-17-92 cluster and its two paraloga in osteosarcoma - reasons and consequences. *Genes Cancer* **2014**, *5*, 56-63, doi:10.18632/genesandcancer.6.
15. Volinia, S.; Calin, G.A.; Liu, C.G.; Ambs, S.; Cimmino, A.; Petrocca, F.; Visone, R.; Iorio, M.; Roldo, C.; Ferracin, M.; et al. A microRNA expression signature of human solid tumors defines cancer gene targets. *Proc Natl Acad Sci U S A* **2006**, *103*, 2257-2261, doi:10.1073/pnas.0510565103.

16. Zhang, X.; Ladd, A.; Dragoescu, E.; Budd, W.T.; Ware, J.L.; Zehner, Z.E. MicroRNA-17-3p is a prostate tumor suppressor in vitro and in vivo, and is decreased in high grade prostate tumors analyzed by laser capture microdissection. *Clin Exp Metastasis* **2009**, *26*, 965-979, doi:10.1007/s10585-009-9287-2.
17. Nam, R.K.; Wallis, C.J.D.; Amemiya, Y.; Benatar, T.; Seth, A. Identification of a Novel MicroRNA Panel Associated with Metastasis Following Radical Prostatectomy for Prostate Cancer. *Anticancer Res* **2018**, *38*, 5027-5034, doi:10.21873/anticanres.12821.
18. Dai, H.; Wang, C.; Yu, Z.; He, D.; Yu, K.; Liu, Y.; Wang, S. MiR-17 Regulates Prostate Cancer Cell Proliferation and Apoptosis Through Inhibiting JAK-STAT3 Signaling Pathway. *Cancer Biother Radiopharm* **2018**, *33*, 103-109, doi:10.1089/cbr.2017.2386.
19. Xu, Z.; Zhang, Y.; Ding, J.; Hu, W.; Tan, C.; Wang, M.; Tang, J.; Xu, Y. miR-17-3p Downregulates Mitochondrial Antioxidant Enzymes and Enhances the Radiosensitivity of Prostate Cancer Cells. *Mol Ther Nucleic Acids* **2018**, *13*, 64-77, doi:10.1016/j.omtn.2018.08.009.
20. Yang, X.; Du, W.W.; Li, H.; Liu, F.; Khorshidi, A.; Rutnam, Z.J.; Yang, B.B. Both mature miR-17-5p and passenger strand miR-17-3p target TIMP3 and induce prostate tumor growth and invasion. *Nucleic Acids Res* **2013**, *41*, 9688-9704, doi:10.1093/nar/gkt680.
21. Gong, A.Y.; Eischeid, A.N.; Xiao, J.; Zhao, J.; Chen, D.; Wang, Z.Y.; Young, C.Y.; Chen, X.M. miR-17-5p targets the p300/CBP-associated factor and modulates androgen receptor transcriptional activity in cultured prostate cancer cells. *BMC Cancer* **2012**, *12*, 492, doi:10.1186/1471-2407-12-492.
22. Dyson, G.; Farran, B.; Bolton, S.; Craig, D.B.; Dombkowski, A.; Beebe-Dimmer, J.L.; Powell, I.J.; Podgorski, I.; Heilbrun, L.K.; Bock, C.H. The extrema of circulating miR-17 are identified as biomarkers for aggressive prostate cancer. *Am J Cancer Res* **2018**, *8*, 2088-2095.
23. Hoey, C.; Ahmed, M.; Fotouhi Ghiam, A.; Vesprini, D.; Huang, X.; Commisso, K.; Commisso, A.; Ray, J.; Fokas, E.; Loblaw, D.A.; et al. Circulating miRNAs as non-invasive biomarkers to predict aggressive prostate cancer after radical prostatectomy. *J Transl Med* **2019**, *17*, 173, doi:10.1186/s12967-019-1920-5.
24. Urabe, F.; Matsuzaki, J.; Yamamoto, Y.; Kimura, T.; Hara, T.; Ichikawa, M.; Takizawa, S.; Aoki, Y.; Niida, S.; Sakamoto, H.; et al. Large-scale Circulating microRNA Profiling for the Liquid Biopsy of Prostate Cancer. *Clin Cancer Res* **2019**, *25*, 3016-3025, doi:10.1158/1078-0432.CCR-18-2849.
25. Wang, X.; Wang, R.; Wu, Z.; Bai, P. Circular RNA ITCH suppressed prostate cancer progression by increasing HOXB13 expression via spongy miR-17-5p. *Cancer Cell Int* **2019**, *19*, 328, doi:10.1186/s12935-019-0994-8.
26. Hu, Y.; Guo, B. Circ-MTO1 correlates with favorable prognosis and inhibits cell proliferation, invasion as well as miR-17-5p expression in prostate cancer. *J Clin Lab Anal* **2020**, *34*, e23086, doi:10.1002/jcla.23086.
27. Xu, Y.; Fang, F.; Zhang, J.; Josson, S.; St Clair, W.H.; St Clair, D.K. miR-17* suppresses tumorigenicity of prostate cancer by inhibiting mitochondrial antioxidant enzymes. *PLoS One* **2010**, *5*, e14356, doi:10.1371/journal.pone.0014356.
28. Dong, P.; Xiong, Y.; Yu, J.; Chen, L.; Tao, T.; Yi, S.; Hanley, S.J.B.; Yue, J.; Watari, H.; Sakuragi, N. Control of PD-L1 expression by miR-140/142/340/383 and oncogenic activation of the OCT4-miR-18a pathway in cervical cancer. *Oncogene* **2018**, *37*, 5257-5268, doi:10.1038/s41388-018-0347-4.
29. Liang, B.; Zhou, C.; Cui, S.; Lu, H.; Xu, R.; Xue, D.; Zou, S.; He, X. Upregulation of miR-18a-5p promotes the proliferation of prostate cancer via inhibiting the expression of SLC40A1. *Pathology - Research and Practice* **2021**, *224*, 153448, doi:https://doi.org/10.1016/j.prp.2021.153448.
30. Hsu, T.I.; Hsu, C.H.; Lee, K.H.; Lin, J.T.; Chen, C.S.; Chang, K.C.; Su, C.Y.; Hsiao, M.; Lu, P.J. MicroRNA-18a is elevated in prostate cancer and promotes tumorigenesis through suppressing STK4 in vitro and in vivo. *Oncogenesis* **2014**, *3*, e99-e99, doi:10.1038/oncsis.2014.12.

31. Ibrahim, N.H.; Abdellateif, M.S.; Kassem, S.H.; Abd El Salam, M.A.; El Gammal, M.M. Diagnostic significance of miR-21, miR-141, miR-18a and miR-221 as novel biomarkers in prostate cancer among Egyptian patients. *Andrologia* **2019**, *51*, e13384, doi:10.1111/and.13384.
32. Al-Kafaji, G.; Al-Naieb, Z.T.; Bakhiet, M. Increased oncogenic microRNA-18a expression in the peripheral blood of patients with prostate cancer: A potential novel non-invasive biomarker. *Oncol Lett* **2016**, *11*, 1201-1206, doi:10.3892/ol.2015.4014.
33. Yang, J.; Hao, T.; Sun, J.; Wei, P.; Zhang, H. Long noncoding RNA GAS5 modulates α-Solanine-induced radiosensitivity by negatively regulating miR-18a in human prostate cancer cells. *Biomed Pharmacother* **2019**, *112*, 108656, doi:10.1016/j.biopha.2019.108656.
34. Zhang, G.; Han, G.; Zhang, X.; Yu, Q.; Li, Z.; Li, Z.; Li, J. Long non-coding RNA FENDRR reduces prostate cancer malignancy by competitively binding miR-18a-5p with RUNX1. *Biomarkers* **2018**, *23*, 435-445, doi:10.1080/1354750x.2018.1443509.
35. Zhao, Y.; Zhang, Q.; Liu, H.; Wang, N.; Zhang, X.; Yang, S. lncRNA PART1, manipulated by transcriptional factor FOXP2, suppresses proliferation and invasion in ESCC by regulating the miR-18a-5p/SOX6 signaling axis. *Oncol Rep* **2021**, *45*, 1118-1132, doi:10.3892/or.2021.7931.
36. Feng, S.; Zhu, X.; Fan, B.; Xie, D.; Li, T.; Zhang, X. miR-19a-3p targets PMEPA1 and induces prostate cancer cell proliferation, migration and invasion. *Mol Med Rep* **2016**, *13*, 4030-4038, doi:10.3892/mmr.2016.5033.
37. Wa, Q.; Li, L.; Lin, H.; Peng, X.; Ren, D.; Huang, Y.; He, P.; Huang, S. Downregulation of miR-19a-3p promotes invasion, migration and bone metastasis via activating TGF-β signaling in prostate cancer. *Oncol Rep* **2018**, *39*, 81-90, doi:10.3892/or.2017.6096.
38. Fu, F.; Wan, X.; Wang, D.; Kong, Z.; Zhang, Y.; Huang, W.; Wang, C.; Wu, H.; Li, Y. MicroRNA-19a acts as a prognostic marker and promotes prostate cancer progression via inhibiting VPS37A expression. *Oncotarget* **2017**, *9*.
39. Lu, K.; Liu, C.; Tao, T.; Zhang, X.; Zhang, L.; Sun, C.; Wang, Y.; Chen, S.; Xu, B.; Chen, M. MicroRNA-19a regulates proliferation and apoptosis of castration-resistant prostate cancer cells by targeting BTG1. *FEBS Lett* **2015**, *589*, 1485-1490, doi:10.1016/j.febslet.2015.04.037.
40. Wang, S.-Y.; Shiboski, S.; Belair, C.D.; Cooperberg, M.R.; Simko, J.P.; Stoppler, H.; Cowan, J.; Carroll, P.R.; Blelloch, R. miR-19, miR-345, miR-519c-5p Serum Levels Predict Adverse Pathology in Prostate Cancer Patients Eligible for Active Surveillance. *PLoS One* **2014**, *9*, e98597, doi:10.1371/journal.pone.0098597.
41. Stuopelyte, K.; Daniunaite, K.; Jankevicius, F.; Jarmalaite, S. Detection of miRNAs in urine of prostate cancer patients. *Medicina (Kaunas)* **2016**, *52*, 116-124, doi:10.1016/j.medici.2016.02.007.
42. Mo, W.; Zhang, J.; Li, X.; Meng, D.; Gao, Y.; Yang, S.; Wan, X.; Zhou, C.; Guo, F.; Huang, Y.; et al. Identification of Novel AR-Targeted MicroRNAs Mediating Androgen Signalling through Critical Pathways to Regulate Cell Viability in Prostate Cancer. *PLoS One* **2013**, *8*, e56592, doi:10.1371/journal.pone.0056592.
43. Qiang, X.F.; Zhang, Z.W.; Liu, Q.; Sun, N.; Pan, L.L.; Shen, J.; Li, T.; Yun, C.; Li, H.; Shi, L.H. miR-20a promotes prostate cancer invasion and migration through targeting ABL2. *J Cell Biochem* **2014**, *115*, 1269-1276, doi:10.1002/jcb.24778.
44. Pesta, M.; Klecka, J.; Kulda, V.; Topolcan, O.; Hora, M.; Eret, V.; Ludvikova, M.; Babjuk, M.; Novak, K.; Stolz, J.; et al. Importance of miR-20a expression in prostate cancer tissue. *Anticancer Res* **2010**, *30*, 3579-3583.
45. Shen, J.; Hruby, G.W.; McKiernan, J.M.; Gurvich, I.; Lipsky, M.J.; Benson, M.C.; Santella, R.M. Dysregulation of circulating microRNAs and prediction of aggressive prostate cancer. *Prostate* **2012**, *72*, 1469-1477, doi:10.1002/pros.22499.
46. Hart, M.; Nolte, E.; Wach, S.; Szczyrba, J.; Taubert, H.; Rau, T.T.; Hartmann, A.; Grasser, F.A.; Wullich, B. Comparative microRNA profiling of prostate carcinomas with increasing tumor stage by deep sequencing. *Mol Cancer Res* **2014**, *12*, 250-263, doi:10.1158/1541-7786.MCR-13-0230.

47. Li, X.; Pan, J.H.; Song, B.; Xiong, E.Q.; Chen, Z.W.; Zhou, Z.S.; Su, Y.P. Suppression of CX43 expression by miR-20a in the progression of human prostate cancer. *Cancer Biol Ther* **2012**, *13*, 890-898, doi:10.4161/cbt.20841.
48. Sylvestre, Y.; De Guire, V.; Querido, E.; Mukhopadhyay, U.K.; Bourdeau, V.; Major, F.; Ferbeyre, G.; Chartrand, P. An E2F/miR-20a autoregulatory feedback loop. *J Biol Chem* **2007**, *282*, 2135-2143, doi:10.1074/jbc.M608939200.
49. Lin, H.M.; Castillo, L.; Mahon, K.L.; Chiam, K.; Lee, B.Y.; Nguyen, Q.; Boyer, M.J.; Stockler, M.R.; Pavlakis, N.; Marx, G.; et al. Circulating microRNAs are associated with docetaxel chemotherapy outcome in castration-resistant prostate cancer. *Br J Cancer* **2014**, *110*, 2462-2471, doi:10.1038/bjc.2014.181.
50. Mohammadi Torbati, P.; Asadi, F.; Fard-Esfahani, P. Circulating miR-20a and miR-26a as Biomarkers in Prostate Cancer. *Asian Pac J Cancer Prev* **2019**, *20*, 1453-1456, doi:10.31557/APJCP.2019.20.5.1453.
51. Daniel, R.; Wu, Q.; Williams, V.; Clark, G.; Guruli, G.; Zehner, Z. A Panel of MicroRNAs as Diagnostic Biomarkers for the Identification of Prostate Cancer. *Int J Mol Sci* **2017**, *18*, doi:10.3390/ijms18061281.
52. Buechner, J.; Tomte, E.; Haug, B.H.; Henriksen, J.R.; Lokke, C.; Flaegstad, T.; Einvik, C. Tumour-suppressor microRNAs let-7 and mir-101 target the proto-oncogene MYCN and inhibit cell proliferation in MYCN-amplified neuroblastoma. *Br J Cancer* **2011**, *105*, 296-303, doi:10.1038/bjc.2011.220.
53. Olive, V.; Bennett, M.J.; Walker, J.C.; Ma, C.; Jiang, I.; Cordon-Cardo, C.; Li, Q.J.; Lowe, S.W.; Hannon, G.J.; He, L. miR-19 is a key oncogenic component of mir-17-92. *Genes Dev* **2009**, *23*, 2839-2849, doi:10.1101/gad.1861409.
54. Fan, Y.; Yin, S.; Hao, Y.; Yang, J.; Zhang, H.; Sun, C.; Ma, M.; Chang, Q.; Xi, J.J. miR-19b promotes tumor growth and metastasis via targeting TP53. *Rna* **2014**, *20*, 765-772, doi:10.1261/rna.043026.113.
55. Zhang, G.; Zhou, H.; Xiao, H.; Liu, Z.; Tian, H.; Zhou, T. MicroRNA-92a functions as an oncogene in colorectal cancer by targeting PTEN. *Dig Dis Sci* **2014**, *59*, 98-107, doi:10.1007/s10620-013-2858-8.
56. Liao, G.; Xiong, H.; Tang, J.; Li, Y.; Liu, Y. MicroRNA-92a Inhibits the Cell Viability and Metastasis of Prostate Cancer by Targeting SOX4. *Technol Cancer Res Treat* **2020**, *19*, 1533033820959354, doi:10.1177/1533033820959354.
57. Xiaoli, Z.; Yawei, W.; Lianna, L.; Haifeng, L.; Hui, Z. Screening of Target Genes and Regulatory Function of miRNAs as Prognostic Indicators for Prostate Cancer. *Med Sci Monit* **2015**, *21*, 3748-3759, doi:10.12659/msm.894670.
58. Zhang, R.; Li, F.; Wang, Y.; Yao, M.; Chi, C. Prognostic value of microRNA-20b expression level in patients with prostate cancer. *Histol Histopathol* **2020**, *35*, 827-831, doi:10.14670/HH-18-216.
59. Tian, L.; Fang, Y.X.; Xue, J.L.; Chen, J.Z. Four microRNAs promote prostate cell proliferation with regulation of PTEN and its downstream signals in vitro. *PLoS One* **2013**, *8*, e75885, doi:10.1371/journal.pone.0075885.
60. Jiao, L.; Deng, Z.; Xu, C.; Yu, Y.; Li, Y.; Yang, C.; Chen, J.; Liu, Z.; Huang, G.; Li, L.C.; et al. miR-663 induces castration-resistant prostate cancer transformation and predicts clinical recurrence. *J Cell Physiol* **2014**, *229*, 834-844, doi:10.1002/jcp.24510.
61. Cho, J.G.; Park, S.; Lim, C.H.; Kim, H.S.; Song, S.Y.; Roh, T.Y.; Sung, J.H.; Suh, W.; Ham, S.J.; Lim, K.H.; et al. ZNF224, Kruppel like zinc finger protein, induces cell growth and apoptosis-resistance by down-regulation of p21 and p53 via miR-663a. *Oncotarget* **2016**, *7*, 31177-31190, doi:10.18632/oncotarget.8870.
62. Dang, Q.; Li, L.; Xie, H.; He, D.; Chen, J.; Song, W.; Chang, L.S.; Chang, H.C.; Yeh, S.; Chang, C. Anti-androgen enzalutamide enhances prostate cancer neuroendocrine (NE) differentiation via altering the infiltrated mast cells --> androgen receptor (AR) --> miRNA32 signals. *Mol Oncol* **2015**, *9*, 1241-1251, doi:10.1016/j.molonc.2015.02.010.
63. Zhu, G.; Chai, J.; Ma, L.; Duan, H.; Zhang, H. Downregulated microRNA-32 expression induced by high glucose inhibits cell cycle progression via PTEN upregulation and Akt inactivation in bone marrow-derived mesenchymal stem cells. *Biochem Biophys Res Commun* **2013**, *433*, 526-531, doi:10.1016/j.bbrc.2013.03.018.
64. Li, J.; Yang, X.; Guan, H.; Mizokami, A.; Keller, E.T.; Xu, X.; Liu, X.; Tan, J.; Hu, L.; Lu, Y.; et al. Exosome-derived microRNAs contribute to prostate cancer chemoresistance. *Int J Oncol* **2016**, *49*, 838-846, doi:10.3892/ijo.2016.3560.

65. Zhang, L.; Li, X.; Chao, Y.; He, R.; Liu, J.; Yuan, Y.; Zhao, W.; Han, C.; Song, X. KLF4, a miR-32-5p targeted gene, promotes cisplatin-induced apoptosis by upregulating BIK expression in prostate cancer. *Cell Commun Signal* **2018**, *16*, 53, doi:10.1186/s12964-018-0270-x.
66. Latonen, L.; Scaravilli, M.; Gillen, A.; Hartikainen, S.; Zhang, F.P.; Ruusuvuori, P.; Kujala, P.; Poutanen, M.; Visakorpi, T. In Vivo Expression of miR-32 Induces Proliferation in Prostate Epithelium. *Am J Pathol* **2017**, *187*, 2546-2557, doi:10.1016/j.ajpath.2017.07.012.
67. Liao, H.; Xiao, Y.; Hu, Y.; Yin, Z.; Liu, L. microRNA-32 induces radioresistance by targeting DAB2IP and regulating autophagy in prostate cancer cells. *Oncol Lett* **2015**, *10*, 2055-2062, doi:10.3892/ol.2015.3551.
68. Song, C.J.; Chen, H.; Chen, L.Z.; Ru, G.M.; Guo, J.J.; Ding, Q.N. The potential of microRNAs as human prostate cancer biomarkers: A meta-analysis of related studies. *J Cell Biochem* **2018**, *119*, 2763-2786, doi:10.1002/jcb.26445.
69. Ambrozkiewicz, F.; Karczmarski, J.; Kulecka, M.; Paziewska, A.; Cybulska, M.; Szymanski, M.; Dobruch, J.; Antoniewicz, A.; Mikula, M.; Ostrowski, J. Challenges in Cancer Biomarker Discovery Exemplified by the Identification of Diagnostic MicroRNAs in Prostate Tissues. *Biomed Res Int* **2020**, *2020*, 9086829, doi:10.1155/2020/9086829.
70. Paziewska, A.; Mikula, M.; Dabrowska, M.; Kulecka, M.; Goryca, K.; Antoniewicz, A.; Dobruch, J.; Borowka, A.; Rutkowski, P.; Ostrowski, J. Candidate diagnostic miRNAs that can detect cancer in prostate biopsy. *Prostate* **2018**, *78*, 178-185, doi:10.1002/pros.23427.
71. Jalava, S.E.; Urbanucci, A.; Latonen, L.; Waltering, K.K.; Sahu, B.; Janne, O.A.; Seppala, J.; Lahdesmaki, H.; Tammela, T.L.; Visakorpi, T. Androgen-regulated miR-32 targets BTG2 and is overexpressed in castration-resistant prostate cancer. *Oncogene* **2012**, *31*, 4460-4471, doi:10.1038/onc.2011.624.
72. Ding, M.; Lin, B.; Li, T.; Liu, Y.; Li, Y.; Zhou, X.; Miao, M.; Gu, J.; Pan, H.; Yang, F.; et al. A dual yet opposite growth-regulating function of miR-204 and its target XRN1 in prostate adenocarcinoma cells and neuroendocrine-like prostate cancer cells. *Oncotarget* **2015**, *6*, 7686-7700, doi:10.18632/oncotarget.3480.
73. Wa, Q.; Huang, S.; Pan, J.; Tang, Y.; He, S.; Fu, X.; Peng, X.; Chen, X.; Yang, C.; Ren, D.; et al. miR-204-5p Represses Bone Metastasis via Inactivating NF-kappaB Signaling in Prostate Cancer. *Mol Ther Nucleic Acids* **2019**, *18*, 567-579, doi:10.1016/j.omtn.2019.09.008.
74. Shu, Y.; Ren, L.; Xie, B.; Liang, Z.; Chen, J. MiR-204 enhances mitochondrial apoptosis in doxorubicin-treated prostate cancer cells by targeting SIRT1/p53 pathway. *Oncotarget* **2017**, *8*, 97313-97322, doi:10.18632/oncotarget.21960.
75. Wu, G.; Wang, J.; Chen, G.; Zhao, X. microRNA-204 modulates chemosensitivity and apoptosis of prostate cancer cells by targeting zinc-finger E-box-binding homeobox 1 (ZEB1). *Am J Transl Res* **2017**, *9*, 3599-3610.
76. Lin, Y.C.; Lin, J.F.; Tsai, T.F.; Chou, K.Y.; Chen, H.E.; Hwang, T.I. Tumor suppressor miRNA-204-5p promotes apoptosis by targeting BCL2 in prostate cancer cells. *Asian J Surg* **2017**, *40*, 396-406, doi:10.1016/j.asjsur.2016.07.001.
77. Fredsoe, J.; Rasmussen, A.K.I.; Mouritzen, P.; Borre, M.; Orntoft, T.; Sorensen, K.D. A five-microRNA model (pCaP) for predicting prostate cancer aggressiveness using cell-free urine. *Int J Cancer* **2019**, *145*, 2558-2567, doi:10.1002/ijc.32296.
78. Koppers-Lalic, D.; Hackenberg, M.; de Menezes, R.; Misovic, B.; Wachalska, M.; Geldof, A.; Zini, N.; de Reijke, T.; Wurdinger, T.; Vis, A.; et al. Noninvasive prostate cancer detection by measuring miRNA variants (isomiRs) in urine extracellular vesicles. *Oncotarget* **2016**, *7*, 22566-22578, doi:10.18632/oncotarget.8124.
79. Lin, Y.; Chen, F.; Shen, L.; Tang, X.; Du, C.; Sun, Z.; Ding, H.; Chen, J.; Shen, B. Biomarker microRNAs for prostate cancer metastasis: screened with a network vulnerability analysis model. *J Transl Med* **2018**, *16*, 134, doi:10.1186/s12967-018-1506-7.
80. Jiang, X.; Guo, S.; Zhang, Y.; Zhao, Y.; Li, X.; Jia, Y.; Xu, Y.; Ma, B. LncRNA NEAT1 promotes docetaxel resistance in prostate cancer by regulating ACSL4 via sponging miR-34a-5p and miR-204-5p. *Cell Signal* **2020**, *65*, 109422, doi:10.1016/j.cellsig.2019.109422.

81. He, C.; Lu, X.; Yang, F.; Qin, L.; Guo, Z.; Sun, Y.; Wu, J. LncRNA UCA1 acts as a sponge of miR-204 to up-regulate CXCR4 expression and promote prostate cancer progression. *Biosci Rep* **2019**, *39*, doi:10.1042/BSR20181465.
82. Zhang, S.; Dong, X.; Ji, T.; Chen, G.; Shan, L. Long non-coding RNA UCA1 promotes cell progression by acting as a competing endogenous RNA of ATF2 in prostate cancer. *Am J Transl Res* **2017**, *9*, 366-375.
83. Todorova, K.; Metodiev, M.V.; Metodieva, G.; Mincheff, M.; Fernandez, N.; Hayrabedyan, S. Micro-RNA-204 Participates in TMPRSS2/ERG Regulation and Androgen Receptor Reprogramming in Prostate Cancer. *Horm Cancer* **2017**, *8*, 28-48, doi:10.1007/s12672-016-0279-9.
84. Panigrahi, G.K.; Ramteke, A.; Birks, D.; Abouzeid Ali, H.E.; Venkataraman, S.; Agarwal, C.; Vibhakar, R.; Miller, L.D.; Agarwal, R.; Abd Elmageed, Z.Y.; et al. Exosomal microRNA profiling to identify hypoxia-related biomarkers in prostate cancer. *Oncotarget* **2018**, *9*, 13894-13910, doi:10.18632/oncotarget.24532.
85. Wei, J.S.; Song, Y.K.; Durinck, S.; Chen, Q.R.; Cheuk, A.T.C.; Tsang, P.; Zhang, Q.; Thiele, C.J.; Slack, A.; Shohet, J.; et al. The MYCN oncogene is a direct target of miR-34a. *Oncogene* **2008**, *27*, 5204-5213, doi:10.1038/onc.2008.154.
86. Choi, Y.J.; Lin, C.P.; Ho, J.J.; He, X.; Okada, N.; Bu, P.; Zhong, Y.; Kim, S.Y.; Bennett, M.J.; Chen, C.; et al. miR-34 miRNAs provide a barrier for somatic cell reprogramming. *Nat Cell Biol* **2011**, *13*, 1353-1360, doi:10.1038/ncb2366.
87. Kashat, M.; Azzouz, L.; Sarkar, S.H.; Kong, D.; Li, Y.; Sarkar, F.H. Inactivation of AR and Notch-1 signaling by miR-34a attenuates prostate cancer aggressiveness. *Am J Transl Res* **2012**, *4*, 432-442.
88. Javeri, A.; Ghaffarpour, M.; Taha, M.F.; Houshmand, M. Downregulation of miR-34a in breast tumors is not associated with either p53 mutations or promoter hypermethylation while it correlates with metastasis. *Med Oncol* **2013**, *30*, 413, doi:10.1007/s12032-012-0413-7.
89. Kong, D.; Heath, E.; Chen, W.; Cher, M.; Powell, I.; Heilbrun, L.; Li, Y.; Ali, S.; Sethi, S.; Hassan, O.; et al. Epigenetic silencing of miR-34a in human prostate cancer cells and tumor tissue specimens can be reversed by BR-DIM treatment. *Am J Transl Res* **2012**, *4*, 14-23.
90. Chen, W.Y.; Liu, S.Y.; Chang, Y.S.; Yin, J.J.; Yeh, H.L.; Mouhieddine, T.H.; Hadadeh, O.; Abou-Kheir, W.; Liu, Y.N. MicroRNA-34a regulates WNT/TCF7 signaling and inhibits bone metastasis in Ras-activated prostate cancer. *Oncotarget* **2015**, *6*, 441-457, doi:10.18632/oncotarget.2690.
91. Zhang, G.; Tian, X.; Li, Y.; Wang, Z.; Li, X.; Zhu, C. miR-27b and miR-34a enhance docetaxel sensitivity of prostate cancer cells through inhibiting epithelial-to-mesenchymal transition by targeting ZEB1. *Biomed Pharmacother* **2018**, *97*, 736-744, doi:10.1016/j.biopha.2017.10.163.
92. Rokavec, M.; Oner, M.G.; Li, H.; Jackstadt, R.; Jiang, L.; Lodygin, D.; Kaller, M.; Horst, D.; Ziegler, P.K.; Schwitalla, S.; et al. IL-6R/STAT3/miR-34a feedback loop promotes EMT-mediated colorectal cancer invasion and metastasis. *J Clin Invest* **2014**, *124*, 1853-1867, doi:10.1172/JCI73531.
93. Duan, K.; Ge, Y.C.; Zhang, X.P.; Wu, S.Y.; Feng, J.S.; Chen, S.L.; Zhang, L.I.; Yuan, Z.H.; Fu, C.H. miR-34a inhibits cell proliferation in prostate cancer by downregulation of SIRT1 expression. *Oncol Lett* **2015**, *10*, 3223-3227, doi:10.3892/ol.2015.3645.
94. Liang, J.; Li, Y.; Daniels, G.; Sfanos, K.; De Marzo, A.; Wei, J.; Li, X.; Chen, W.; Wang, J.; Zhong, X.; et al. LEF1 Targeting EMT in Prostate Cancer Invasion Is Regulated by miR-34a. *Mol Cancer Res* **2015**, *13*, 681-688, doi:10.1158/1541-7786.MCR-14-0503.
95. Liao, H.; Xiao, Y.; Hu, Y.; Yin, Z.; Liu, L.; Kang, X.; Chen, Y. Methylation-induced silencing of miR-34a enhances chemoresistance by directly upregulating ATG4B-induced autophagy through AMPK/mTOR pathway in prostate cancer. *Oncol Rep* **2016**, *35*, 64-72, doi:10.3892/or.2015.4331.

96. Liu, X.; Luo, X.; Wu, Y.; Xia, D.; Chen, W.; Fang, Z.; Deng, J.; Hao, Y.; Yang, X.; Zhang, T.; et al. MicroRNA-34a Attenuates Paclitaxel Resistance in Prostate Cancer Cells via Direct Suppression of JAG1/Notch1 Axis. *Cell Physiol Biochem* **2018**, *50*, 261-276, doi:10.1159/000494004.
97. Corcoran, C.; Rani, S.; O'Driscoll, L. miR-34a is an intracellular and exosomal predictive biomarker for response to docetaxel with clinical relevance to prostate cancer progression. *Prostate* **2014**, *74*, 1320-1334, doi:10.1002/pros.22848.
98. Ma, Y.; Fan, B.; Ren, Z.; Liu, B.; Wang, Y. Long noncoding RNA DANCR contributes to docetaxel resistance in prostate cancer through targeting the miR-34a-5p/JAG1 pathway. *Onco Targets Ther* **2019**, *12*, 5485-5497, doi:10.2147/OTT.S197009.
99. Ma, E.; Wang, Q.; Li, J.; Zhang, X.; Guo, Z.; Yang, X. LINC01006 facilitates cell proliferation, migration and invasion in prostate cancer through targeting miR-34a-5p to up-regulate DAAM1. *Cancer Cell Int* **2020**, *20*, 515, doi:10.1186/s12935-020-01577-1.
100. Li, N.; Zhang, L.Y.; Qiao, Y.H.; Song, R.J. Long noncoding RNA LINC00662 functions as miRNA sponge to promote the prostate cancer tumorigenesis through targeting miR-34a. *Eur Rev Med Pharmacol Sci* **2019**, *23*, 3688-3698, doi:10.26355/eurrev_201905_17792.
101. Lodygin, D.; Tarasov, V.; Epanchintsev, A.; Berking, C.; Knyazeva, T.; Körner, H.; Knyazev, P.; Diebold, J.; Hermeking, H. Inactivation of miR-34a by aberrant CpG methylation in multiple types of cancer. *Cell Cycle* **2008**, *7*, 2591-2600, doi:10.4161/cc.7.16.6533.
102. Zheng, C.; Yinghao, S.; Li, J. MiR-221 expression affects invasion potential of human prostate carcinoma cell lines by targeting DVL2. *Med Oncol* **2012**, *29*, 815-822, doi:10.1007/s12032-011-9934-8.
103. Garofalo, M.; Di Leva, G.; Romano, G.; Nuovo, G.; Suh, S.-S.; Ngankeu, A.; Taccioli, C.; Pichiorri, F.; Alder, H.; Secchiero, P.; et al. miR-221&222 Regulate TRAIL Resistance and Enhance Tumorigenicity through PTEN and TIMP3 Downregulation. *Cancer Cell* **2009**, *16*, 498-509, doi:https://doi.org/10.1016/j.ccr.2009.10.014.
104. Lupini, L.; Bassi, C.; Ferracin, M.; Bartonicek, N.; D'Abundo, L.; Zagatti, B.; Callegari, E.; Musa, G.; Moshiri, F.; Gramantieri, L.; et al. miR-221 affects multiple cancer pathways by modulating the level of hundreds messenger RNAs. *Front Genet* **2013**, *4*, doi:10.3389/fgene.2013.00064.
105. Kiener, M.; Chen, L.; Krebs, M.; Grosjean, J.; Klima, I.; Kalogirou, C.; Riedmiller, H.; Kneitz, B.; Thalmann, G.N.; Snaar-Jagalska, E.; et al. miR-221-5p regulates proliferation and migration in human prostate cancer cells and reduces tumor growth in vivo. *BMC Cancer* **2019**, *19*, 627, doi:10.1186/s12885-019-5819-6.
106. Xuan, H.; Xue, W.; Pan, J.; Sha, J.; Dong, B.; Huang, Y. Downregulation of miR-221, -30d, and -15a contributes to pathogenesis of prostate cancer by targeting Bmi-1. *Biochemistry (Moscow)* **2015**, *80*, 276-283, doi:10.1134/S0006297915030037.
107. Valera, V.A.; Parra-Medina, R.; Walter, B.A.; Pinto, P.; Merino, M.J. microRNA Expression Profiling in Young Prostate Cancer Patients. *J Cancer* **2020**, *11*, 4106-4114, doi:10.7150/jca.37842.
108. Sun, T.; Du, S.-Y.; Armenia, J.; Qu, F.; Fan, J.; Wang, X.; Fei, T.; Komura, K.; Liu, S.X.; Lee, G.-S.M.; et al. Expression of lncRNA MIR222HG co-transcribed from the miR-221/222 gene promoter facilitates the development of castration-resistant prostate cancer. *Oncogenesis* **2018**, *7*, 30, doi:10.1038/s41389-018-0039-5.
109. Krebs, M.; Behrmann, C.; Kalogirou, C.; Sokolakis, I.; Kneitz, S.; Kruithof-de Julio, M.; Zoni, E.; Rech, A.; Schilling, B.; Kubler, H.; et al. miR-221 Augments TRAIL-Mediated Apoptosis in Prostate Cancer Cells by Inducing Endogenous TRAIL Expression and Targeting the Functional Repressors SOCS3 and PIK3R1. *Biomed Res Int* **2019**, *2019*, 6392748, doi:10.1155/2019/6392748.

110. Kurul, N.O.; Ates, F.; Yilmaz, I.; Narli, G.; Yesildal, C.; Senkul, T. The association of let-7c, miR-21, miR-145, miR-182, and miR-221 with clinicopathologic parameters of prostate cancer in patients diagnosed with low-risk disease. *Prostate* **2019**, *79*, 1125-1132, doi:10.1002/pros.23825.
111. Pashaei, E.; Ahmady, M.; Ozen, M.; Aydin, N. Meta-analysis of miRNA expression profiles for prostate cancer recurrence following radical prostatectomy. *PLoS One* **2017**, *12*, e0179543, doi:10.1371/journal.pone.0179543.
112. Coarfa, C.; Fiskus, W.; Eedunuri, V.K.; Rajapakshe, K.; Foley, C.; Chew, S.A.; Shah, S.S.; Geng, C.; Shou, J.; Mohamed, J.S.; et al. Comprehensive proteomic profiling identifies the androgen receptor axis and other signaling pathways as targets of microRNAs suppressed in metastatic prostate cancer. *Oncogene* **2016**, *35*, 2345-2356, doi:10.1038/onc.2015.295.
113. Varambally, S.; Cao, Q.; Mani, R.S.; Shankar, S.; Wang, X.; Ateeq, B.; Laxman, B.; Cao, X.; Jing, X.; Ramnarayanan, K.; et al. Genomic loss of microRNA-101 leads to overexpression of histone methyltransferase EZH2 in cancer. *Science* **2008**, *322*, 1695-1699, doi:10.1126/science.1165395.
114. Li, K.; Liu, C.; Zhou, B.; Bi, L.; Huang, H.; Lin, T.; Xu, K. Role of EZH2 in the Growth of Prostate Cancer Stem Cells Isolated from LNCaP Cells. *Int J Mol Sci* **2013**, *14*, 11981-11993.
115. Gu, Z.; You, Z.; Yang, Y.; Ding, R.; Wang, M.; Pu, J.; Chen, J. Inhibition of MicroRNA miR-101-3p on prostate cancer progression by regulating Cullin 4B (CUL4B) and PI3K/AKT/mTOR signaling pathways. *Bioengineered* **2021**, *12*, 4719-4735, doi:10.1080/21655979.2021.1949513.
116. Lin, S.-C.; Kao, C.-Y.; Lee, H.-J.; Creighton, C.J.; Ittmann, M.M.; Tsai, S.-J.; Tsai, S.Y.; Tsai, M.-J. Dysregulation of miRNAs-COUP-TFII-FOXM1-CENPF axis contributes to the metastasis of prostate cancer. *Nat Commun* **2016**, *7*, 11418, doi:10.1038/ncomms11418.
117. Antognelli, C.; Cecchetti, R.; Riuzzi, F.; Peirce, M.J.; Talesa, V.N. Glyoxalase 1 sustains the metastatic phenotype of prostate cancer cells via EMT control. *Journal of Cellular and Molecular Medicine* **2018**, *22*, 2865-2883, doi:https://doi.org/10.1111/jcmm.13581.
118. Hao, Y.; Gu, X.; Zhao, Y.; Greene, S.; Sha, W.; Smoot, D.T.; Califano, J.; Wu, T.C.; Pang, X. Enforced expression of miR-101 inhibits prostate cancer cell growth by modulating the COX-2 pathway in vivo. *Cancer Prev Res (Phila)* **2011**, *4*, 1073-1083, doi:10.1158/1940-6207.Capr-10-0333.
119. Li, P.; You, S.; Nguyen, C.; Wang, Y.; Kim, J.; Sirohi, D.; Ziembiec, A.; Luthringer, D.; Lin, S.C.; Daskivich, T.; et al. Genes involved in prostate cancer progression determine MRI visibility. *Theranostics* **2018**, *8*, 1752-1765, doi:10.7150/thno.23180.
120. Duca, R.B.; Massillo, C.; Dalton, G.N.; Farré, P.L.; Graña, K.D.; Gardner, K.; De Siervi, A. MiR-19b-3p and miR-101-3p as potential biomarkers for prostate cancer diagnosis and prognosis. *Am J Cancer Res* **2021**, *11*, 2802-2820.
121. Srivastava, A.; Goldberger, H.; Dimtchev, A.; Marian, C.; Soldin, O.; Li, X.; Collins, S.P.; Suy, S.; Kumar, D. Circulatory miR-628-5p is downregulated in prostate cancer patients. *Tumour Biol* **2014**, *35*, 4867-4873, doi:10.1007/s13277-014-1638-1.
122. Watahiki, A.; Wang, Y.; Morris, J.; Dennis, K.; O'Dwyer, H.M.; Gleave, M.; Gout, P.W.; Wang, Y. MicroRNAs Associated with Metastatic Prostate Cancer. *PLoS One* **2011**, *6*, e24950, doi:10.1371/journal.pone.0024950.
123. Chen, J.H.; Tong, W.; Pu, X.F.; Wang, J.Z. Long noncoding RNA CRNDE promotes proliferation, migration and invasion in prostate cancer through miR-101/Rap1A. *Neoplasma* **2020**, *67*, 584-594, doi:10.4149/neo_2020_190621N534.
124. Lo, U.G.; Pong, R.C.; Yang, D.; Gandee, L.; Hernandez, E.; Dang, A.; Lin, C.J.; Santoyo, J.; Ma, S.; Sonavane, R.; et al. IFNgamma-Induced IFIT5 Promotes Epithelial-to-Mesenchymal Transition in Prostate Cancer via miRNA Processing. *Cancer Res* **2019**, *79*, 1098-1112, doi:10.1158/0008-5472.CAN-18-2207.

125. Cao, P.; Deng, Z.; Wan, M.; Huang, W.; Cramer, S.D.; Xu, J.; Lei, M.; Sui, G. MicroRNA-101 negatively regulates Ezh2 and its expression is modulated by androgen receptor and HIF-1α/HIF-1β. *Mol Cancer* **2010**, *9*, 108, doi:10.1186/1476-4598-9-108.
126. Wu, X.; Bhayani, M.K.; Dodge, C.T.; Nicoloso, M.S.; Chen, Y.; Yan, X.; Adachi, M.; Thomas, L.; Galer, C.E.; Jiffar, T.; et al. Coordinated targeting of the EGFR signaling axis by microRNA-27a*. *Oncotarget* **2013**, *4*, 1388-1398, doi:10.18632/oncotarget.1239.
127. Maqbool, R.; Lone, S.N.; Ul Hussain, M. Post-transcriptional regulation of the tumor suppressor p53 by a novel miR-27a, with implications during hypoxia and tumorigenesis. *Biochem J* **2016**, *473*, 3597-3610, doi:10.1042/bcj20160359.
128. Gao, W.; Hong, Z.; Huang, H.; Zhu, A.; Lin, S.; Cheng, C.; Zhang, X.; Zou, G.; Shi, Z. miR-27a in serum acts as biomarker for prostate cancer detection and promotes cell proliferation by targeting Sprouty2. *Oncol Lett* **2018**, *16*, 5291-5298, doi:10.3892/ol.2018.9274.
129. Wan, X.; Huang, W.; Yang, S.; Zhang, Y.; Zhang, P.; Kong, Z.; Li, T.; Wu, H.; Jing, F.; Li, Y. Androgen-induced miR-27A acted as a tumor suppressor by targeting MAP2K4 and mediated prostate cancer progression. *Int J Biochem Cell Biol* **2016**, *79*, 249-260, doi:10.1016/j.biocel.2016.08.043.
130. Barros-Silva, D.; Costa-Pinheiro, P.; Duarte, H.; Sousa, E.J.; Evangelista, A.F.; Graça, I.; Carneiro, I.; Martins, A.T.; Oliveira, J.; Carvalho, A.L.; et al. MicroRNA-27a-5p regulation by promoter methylation and MYC signaling in prostate carcinogenesis. *Cell Death Dis* **2018**, *9*, 167, doi:10.1038/s41419-017-0241-y.
131. Ku, A.; Fredsøe, J.; Sørensen, K.D.; Borre, M.; Evander, M.; Laurell, T.; Lilja, H.; Ceder, Y. High-Throughput and Automated Acoustic Trapping of Extracellular Vesicles to Identify microRNAs With Diagnostic Potential for Prostate Cancer. *Front Oncol* **2021**, *11*, doi:10.3389/fonc.2021.631021.
132. Cao, Z.; Xu, L.; Zhao, S. Exosome-derived miR-27a produced by PSC-27 cells contributes to prostate cancer chemoresistance through p53. *Biochem Biophys Res Commun* **2019**, *515*, 345-351, doi:10.1016/j.bbrc.2019.05.120.
133. Lyu, J.; Zhao, L.; Wang, F.; Ji, J.; Cao, Z.; Xu, H.; Shi, X.; Zhu, Y.; Zhang, C.; Guo, F.; et al. Discovery and Validation of Serum MicroRNAs as Early Diagnostic Biomarkers for Prostate Cancer in Chinese Population. *Biomed Res Int* **2019**, *2019*, 9306803, doi:10.1155/2019/9306803.
134. Fletcher, C.E.; Dart, D.A.; Sita-Lumsden, A.; Cheng, H.; Rennie, P.S.; Bevan, C.L. Androgen-regulated processing of the oncomir miR-27a, which targets Prohibitin in prostate cancer. *Hum Mol Genet* **2012**, *21*, 3112-3127, doi:10.1093/hmg/dds139.
135. Sun, F.; Wu, K.; Yao, Z.; Mu, X.; Zheng, Z.; Sun, M.; Wang, Y.; Liu, Z.; Zhu, Y. Long Noncoding RNA PVT1 Promotes Prostate Cancer Metastasis by Increasing NOP2 Expression via Targeting Tumor Suppressor MicroRNAs. *Onco Targets Ther* **2020**, *13*, 6755-6765, doi:10.2147/ott.S242441.
136. Cui, X.; Piao, C.; Lv, C.; Lin, X.; Zhang, Z.; Liu, X. ZNFX1 anti-sense RNA 1 promotes the tumorigenesis of prostate cancer by regulating c-Myc expression via a regulatory network of competing endogenous RNAs. *Cell Mol Life Sci* **2020**, *77*, 1135-1152, doi:10.1007/s00018-019-03226-x.
137. Wu, J.; Zheng, C.; Fan, Y.; Zeng, C.; Chen, Z.; Qin, W.; Zhang, C.; Zhang, W.; Wang, X.; Zhu, X.; et al. Downregulation of microRNA-30 facilitates podocyte injury and is prevented by glucocorticoids. *J Am Soc Nephrol* **2014**, *25*, 92-104, doi:10.1681/ASN.2012111101.
138. Kumar, B.; Khaleghzadegan, S.; Mears, B.; Hatano, K.; Kudrolli, T.A.; Chowdhury, W.H.; Yeater, D.B.; Ewing, C.M.; Luo, J.; Isaacs, W.B.; et al. Identification of miR-30b-3p and miR-30d-5p as direct regulators of androgen receptor signaling in prostate cancer by complementary functional microRNA library screening. *Oncotarget* **2016**, *7*, 72593-72607, doi:10.18632/oncotarget.12241.

139. Ren, Q.; Liang, J.; Wei, J.; Basturk, O.; Wang, J.; Daniels, G.; Gellert, L.L.; Li, Y.; Shen, Y.; Osman, I.; et al. Epithelial and stromal expression of miRNAs during prostate cancer progression. *Am J Transl Res* **2014**, *6*, 329-339.
140. Fredsøe, J.; Rasmussen, A.K.I.; Thomsen, A.R.; Mouritzen, P.; Høyer, S.; Borre, M.; Ørntoft, T.F.; Sørensen, K.D. Diagnostic and Prognostic MicroRNA Biomarkers for Prostate Cancer in Cell-free Urine. *Eur Urol Focus* **2018**, *4*, 825-833, doi:10.1016/j.euf.2017.02.018.
141. Zhao, Z.; Weickmann, S.; Jung, M.; Lein, M.; Kilic, E.; Stephan, C.; Erbersdobler, A.; Fendler, A.; Jung, K. A Novel Predictor Tool of Biochemical Recurrence after Radical Prostatectomy Based on a Five-MicroRNA Tissue Signature. *Cancers (Basel)* **2019**, *11*, doi:10.3390/cancers11101603.
142. Chen, W.; Yao, G.; Zhou, K. miR-103a-2-5p/miR-30c-1-3p inhibits the progression of prostate cancer resistance to androgen ablation therapy via targeting androgen receptor variant 7. *J Cell Biochem* **2019**, *120*, 14055-14064, doi:https://doi.org/10.1002/jcb.28680.
143. Esposito, F.; Tornincasa, M.; Pallante, P.; Federico, A.; Borbone, E.; Pierantoni, G.M.; Fusco, A. Down-regulation of the miR-25 and miR-30d contributes to the development of anaplastic thyroid carcinoma targeting the polycomb protein EZH2. *J Clin Endocrinol Metab* **2012**, *97*, E710-718, doi:10.1210/jc.2011-3068.
144. Kumar, M.; Lu, Z.; Takwi, A.A.; Chen, W.; Callander, N.S.; Ramos, K.S.; Young, K.H.; Li, Y. Negative regulation of the tumor suppressor p53 gene by microRNAs. *Oncogene* **2011**, *30*, 843-853, doi:10.1038/onc.2010.457.
145. Song, Y.; Song, C.; Yang, S. Tumor-Suppressive Function of miR-30d-5p in Prostate Cancer Cell Proliferation and Migration by Targeting NT5E. *Cancer Biother Radiopharm* **2018**, *33*, 203-211, doi:10.1089/cbr.2018.2457.
146. Barceló, M.; Castells, M.; Pérez-Riba, M.; Bassas, L.; Vigués, F.; Larriba, S. Seminal plasma microRNAs improve diagnosis/prognosis of prostate cancer in men with moderately altered prostate-specific antigen. *Am J Transl Res* **2020**, *12*, 2041-2051.
147. Fuse, M.; Kojima, S.; Enokida, H.; Chiyomaru, T.; Yoshino, H.; Nohata, N.; Kinoshita, T.; Sakamoto, S.; Naya, Y.; Nakagawa, M.; et al. Tumor suppressive microRNAs (miR-222 and miR-31) regulate molecular pathways based on microRNA expression signature in prostate cancer. *Journal of Human Genetics* **2012**, *57*, 691-699, doi:10.1038/jhg.2012.95.
148. Tsuchiyama, K.; Ito, H.; Taga, M.; Naganuma, S.; Oshinoya, Y.; Nagano, K.; Yokoyama, O.; Itoh, H. Expression of microRNAs associated with Gleason grading system in prostate cancer: miR-182-5p is a useful marker for high grade prostate cancer. *Prostate* **2013**, *73*, 827-834, doi:10.1002/pros.22626.
149. Bian, X.; Shen, Y.; Zhang, G.; Gu, C.; Cai, Y.; Wang, C.; Zhu, Y.; Zhu, Y.; Zhang, H.; Dai, B.; et al. Expression of Dicer and Its Related MiRNAs in the Progression of Prostate Cancer. *PLoS One* **2015**, *10*, e0120159, doi:10.1371/journal.pone.0120159.
150. Zhao, J.; Xu, H.; Duan, Z.; Chen, X.; Ao, Z.; Chen, Y.; Ruan, Y.; Ni, M. miR-31-5p Regulates 14-3-3 ε to Inhibit Prostate Cancer 22RV1 Cell Survival and Proliferation via PI3K/AKT/Bcl-2 Signaling Pathway. *Cancer Manag Res* **2020**, *12*, 6679-6694, doi:10.2147/cmar.S247780.
151. Lin, P.C.; Chiu, Y.L.; Banerjee, S.; Park, K.; Mosquera, J.M.; Giannopoulou, E.; Alves, P.; Tewari, A.K.; Gerstein, M.B.; Beltran, H.; et al. Epigenetic repression of miR-31 disrupts androgen receptor homeostasis and contributes to prostate cancer progression. *Cancer Res* **2013**, *73*, 1232-1244, doi:10.1158/0008-5472.Can-12-2968.
152. Bhatnagar, N.; Li, X.; Padi, S.K.R.; Zhang, Q.; Tang, M.s.; Guo, B. Downregulation of miR-205 and miR-31 confers resistance to chemotherapy-induced apoptosis in prostate cancer cells. *Cell Death Dis* **2010**, *1*, e105-e105, doi:10.1038/cddis.2010.85.
153. Daniunaite, K.; Dubikaityte, M.; Gibas, P.; Bakavicius, A.; Rimantas Lazutka, J.; Ulys, A.; Jankevicius, F.; Jarmalaite, S. Clinical significance of miRNA host gene promoter methylation in prostate cancer. *Hum Mol Genet* **2017**, *26*, 2451-2461, doi:10.1093/hmg/ddx138.

154. Lekchnov, E.A.; Amelina, E.V.; Bryzgunova, O.E.; Zaporozhchenko, I.A.; Konoshenko, M.Y.; Yarmoschuk, S.V.; Murashov, I.S.; Pashkovskaya, O.A.; Gorizkii, A.M.; Zheravin, A.A.; et al. Searching for the Novel Specific Predictors of Prostate Cancer in Urine: The Analysis of 84 miRNA Expression. *Int J Mol Sci* **2018**, *19*, 4088.
155. Fletcher, C.E.; Sulpice, E.; Combe, S.; Shibakawa, A.; Leach, D.A.; Hamilton, M.P.; Chrysostomou, S.L.; Sharp, A.; Welti, J.; Yuan, W.; et al. Androgen receptor-modulatory microRNAs provide insight into therapy resistance and therapeutic targets in advanced prostate cancer. *Oncogene* **2019**, *38*, 5700-5724, doi:10.1038/s41388-019-0823-5.
156. Liu, B.; Sun, Y.; Tang, M.; Liang, C.; Huang, C.P.; Niu, Y.; Wang, Z.; Chang, C. The miR-361-3p increases enzalutamide (Enz) sensitivity via targeting the ARv7 and MKNK2 to better suppress the Enz-resistant prostate cancer. *Cell Death Dis* **2020**, *11*, 807, doi:10.1038/s41419-020-02932-w.
157. Liu, D.; Tao, T.; Xu, B.; Chen, S.; Liu, C.; Zhang, L.; Lu, K.; Huang, Y.; Jiang, L.; Zhang, X.; et al. MiR-361-5p acts as a tumor suppressor in prostate cancer by targeting signal transducer and activator of transcription-6(STAT6). *Biochem Biophys Res Commun* **2014**, *445*, 151-156, doi:10.1016/j.bbrc.2014.01.140.
158. Zhu, J.; Wang, S.; Zhang, W.; Qiu, J.; Shan, Y.; Yang, D.; Shen, B. Screening key microRNAs for castration-resistant prostate cancer based on miRNA/mRNA functional synergistic network. *Oncotarget* **2015**, *6*, 43819-43830, doi:10.18632/oncotarget.6102.
159. Ju, G.; Zhu, Y.; Du, T.; Cao, W.; Lin, J.; Li, C.; Xu, D.; Wang, Z. MiR-197 Inhibitor Loaded AbCD133@MSNs@GNR Affects the Development of Prostate Cancer Through Targeting ITGAV. *Frontiers in Cell and Developmental Biology* **2021**, *9*, doi:10.3389/fcell.2021.646884.
160. Ebron, J.S.; Shankar, E.; Singh, J.; Sikand, K.; Weyman, C.M.; Gupta, S.; Lindner, D.J.; Liu, X.; Campbell, M.J.; Shukla, G.C. MiR-644a Disrupts Oncogenic Transformation and Warburg Effect by Direct Modulation of Multiple Genes of Tumor-Promoting Pathways. *Cancer Res* **2019**, *79*, 1844-1856, doi:10.1158/0008-5472.Can-18-2993.
161. Qu, H.W.; Jin, Y.; Cui, Z.L.; Jin, X.B. MicroRNA-373-3p inhibits prostate cancer progression by targeting AKT1. *Eur Rev Med Pharmacol Sci* **2018**, *22*, 6252-6259, doi:10.26355/eurrev_201810_16032.
162. Walter, B.A.; Valera, V.A.; Pinto, P.A.; Merino, M.J. Comprehensive microRNA Profiling of Prostate Cancer. *J Cancer* **2013**, *4*, 350-357, doi:10.7150/jca.6394.
163. Qiu, X.; Zhu, J.; Sun, Y.; Fan, K.; Yang, D.R.; Li, G.; Yang, G.; Chang, C. TR4 nuclear receptor increases prostate cancer invasion via decreasing the miR-373-3p expression to alter TGFβR2/p-Smad3 signals. *Oncotarget* **2015**, *6*, 15397-15409, doi:10.18632/oncotarget.3778.
164. Yang, K.; Handorean, A.M.; Iczkowski, K.A. MicroRNAs 373 and 520c are downregulated in prostate cancer, suppress CD44 translation and enhance invasion of prostate cancer cells in vitro. *Int J Clin Exp Pathol* **2009**, *2*, 361-369.
165. Zhang, G.; Liu, Z.; Xu, H.; Yang, Q. miR-409-3p suppresses breast cancer cell growth and invasion by targeting Akt1. *Biochem Biophys Res Commun* **2016**, *469*, 189-195, doi:10.1016/j.bbrc.2015.11.099.
166. Josson, S.; Gururajan, M.; Hu, P.; Shao, C.; Chu, G.Y.; Zhau, H.E.; Liu, C.; Lao, K.; Lu, C.L.; Lu, Y.T.; et al. miR-409-3p/-5p promotes tumorigenesis, epithelial-to-mesenchymal transition, and bone metastasis of human prostate cancer. *Clin Cancer Res* **2014**, *20*, 4636-4646, doi:10.1158/1078-0432.Ccr-14-0305.
167. Josson, S.; Gururajan, M.; Sung, S.Y.; Hu, P.; Shao, C.; Zhau, H.E.; Liu, C.; Lichterman, J.; Duan, P.; Li, Q.; et al. Stromal fibroblast-derived miR-409 promotes epithelial-to-mesenchymal transition and prostate tumorigenesis. *Oncogene* **2015**, *34*, 2690-2699, doi:10.1038/onc.2014.212.
168. Fredsoe, J.; Rasmussen, A.K.I.; Mouritzen, P.; Bjerre, M.T.; Ostergren, P.; Fode, M.; Borre, M.; Sorensen, K.D. Profiling of Circulating microRNAs in Prostate Cancer Reveals Diagnostic Biomarker Potential. *Diagnostics (Basel)* **2020**, *10*, doi:10.3390/diagnostics10040188.

169. Nguyen, H.C.; Xie, W.; Yang, M.; Hsieh, C.L.; Drouin, S.; Lee, G.S.; Kantoff, P.W. Expression differences of circulating microRNAs in metastatic castration resistant prostate cancer and low-risk, localized prostate cancer. *Prostate* **2013**, *73*, 346-354, doi:10.1002/pros.22572.
170. Yu, Q.; Li, P.; Weng, M.; Wu, S.; Zhang, Y.; Chen, X.; Zhang, Q.; Shen, G.; Ding, X.; Fu, S. Nano-Vesicles are a Potential Tool to Monitor Therapeutic Efficacy of Carbon Ion Radiotherapy in Prostate Cancer. *J Biomed Nanotechnol* **2018**, *14*, 168-178, doi:10.1166/jbn.2018.2503.
171. Luo, J.; Wang, K.; Yeh, S.; Sun, Y.; Liang, L.; Xiao, Y.; Xu, W.; Niu, Y.; Cheng, L.; Maity, S.N.; et al. LncRNA-p21 alters the antiandrogen enzalutamide-induced prostate cancer neuroendocrine differentiation via modulating the EZH2/STAT3 signaling. *Nat Commun* **2019**, *10*, 2571, doi:10.1038/s41467-019-09784-9.
172. Işın, M.; Uysaler, E.; Özgür, E.; Köseoğlu, H.; Şanlı, Ö.; Yücel Ö, B.; Gezer, U.; Dalay, N. Exosomal lncRNA-p21 levels may help to distinguish prostate cancer from benign disease. *Front Genet* **2015**, *6*, 168, doi:10.3389/fgene.2015.00168.
173. Liu, B.; Jiang, H.-Y.; Yuan, T.; Luo, J.; Zhou, W.-D.; Jiang, Q.-Q.; Wu, D. Enzalutamide-Induced Upregulation of PCAT6 Promotes Prostate Cancer Neuroendocrine Differentiation by Regulating miR-326/HNRNPA2B1 Axis. *Front Oncol* **2021**, *11*, doi:10.3389/fonc.2021.650054.
174. Lang, C.; Yin, C.; Lin, K.; Li, Y.; Yang, Q.; Wu, Z.; Du, H.; Ren, D.; Dai, Y.; Peng, X. m6A modification of lncRNA PCAT6 promotes bone metastasis in prostate cancer through IGF2BP2-mediated IGF1R mRNA stabilization. *Clinical and Translational Medicine* **2021**, *11*, e426, doi:https://doi.org/10.1002/ctm2.426.
175. Ostano, P.; Mello-Grand, M.; Sesia, D.; Gregnanin, I.; Peraldo-Neia, C.; Guana, F.; Jachetti, E.; Farsetti, A.; Chiorino, G. Gene Expression Signature Predictive of Neuroendocrine Transformation in Prostate Adenocarcinoma. *Int J Mol Sci* **2020**, *21*, 1078.
176. Xiang, S.; Zou, P.; Tang, Q.; Zheng, F.; Wu, J.; Chen, Z.; Hann, S.S. HOTAIR-mediated reciprocal regulation of EZH2 and DNMT1 contribute to polyphyllin I-inhibited growth of castration-resistant prostate cancer cells in vitro and in vivo. *Biochimica et Biophysica Acta (BBA) - General Subjects* **2018**, *1862*, 589-599, doi:https://doi.org/10.1016/j.bbagen.2017.12.001.
177. Chang, Y.-T.; Lin, T.-P.; Tang, J.-T.; Campbell, M.; Luo, Y.-L.; Lu, S.-Y.; Yang, C.-P.; Cheng, T.-Y.; Chang, C.-H.; Liu, T.-T.; et al. HOTAIR is a REST-regulated lncRNA that promotes neuroendocrine differentiation in castration resistant prostate cancer. *Cancer Lett* **2018**, *433*, 43-52, doi:https://doi.org/10.1016/j.canlet.2018.06.029.
178. Li, T.; Liu, N.; Gao, Y.; Quan, Z.; Hao, Y.; Yu, C.; Li, L.; Yuan, M.; Niu, L.; Luo, C.; et al. Long noncoding RNA HOTAIR regulates the invasion and metastasis of prostate cancer by targeting hepaCAM. *Br J Cancer* **2021**, *124*, 247-258, doi:10.1038/s41416-020-01091-1.
179. Zhang, A.; Zhao, J.C.; Kim, J.; Fong, K.W.; Yang, Y.A.; Chakravarti, D.; Mo, Y.Y.; Yu, J. LncRNA HOTAIR Enhances the Androgen-Receptor-Mediated Transcriptional Program and Drives Castration-Resistant Prostate Cancer. *Cell Rep* **2015**, *13*, 209-221, doi:10.1016/j.celrep.2015.08.069.
180. Wang, N.; Jiang, Y.; Lv, S.; Wen, H.; Wu, D.; Wei, Q.; Dang, Q. HOTAIR expands the population of prostatic cancer stem-like cells and causes Docetaxel resistance via activating STAT3 signaling. *Aging (Albany NY)* **2020**, *12*, 12771-12782, doi:10.18632/aging.103188.
181. Ling, Z.; Wang, X.; Tao, T.; Zhang, L.; Guan, H.; You, Z.; Lu, K.; Zhang, G.; Chen, S.; Wu, J.; et al. Involvement of aberrantly activated HOTAIR/EZH2/miR-193a feedback loop in progression of prostate cancer. *Journal of Experimental & Clinical Cancer Research* **2017**, *36*, 159, doi:10.1186/s13046-017-0629-7.
182. Wang, D.; Ding, L.; Wang, L.; Zhao, Y.; Sun, Z.; Karnes, R.J.; Zhang, J.; Huang, H. LncRNA MALAT1 enhances oncogenic activities of EZH2 in castration-resistant prostate cancer. *Oncotarget* **2015**, *6*.

183. Hao, T.; Wang, Z.; Yang, J.; Zhang, Y.; Shang, Y.; Sun, J. MALAT1 knockdown inhibits prostate cancer progression by regulating miR-140/BIRC6 axis. *Biomedicine & Pharmacotherapy* **2020**, *123*, 109666, doi:https://doi.org/10.1016/j.biopha.2019.109666.
184. Chang, J.; Xu, W.; Du, X.; Hou, J. MALAT1 silencing suppresses prostate cancer progression by upregulating miR-1 and downregulating KRAS. *Onco Targets Ther* **2018**, *11*, 3461-3473, doi:10.2147/ott.S164131.
185. Xue, D.; Lu, H.; Xu, H.-Y.; Zhou, C.-X.; He, X.-Z. Long noncoding RNA MALAT1 enhances the docetaxel resistance of prostate cancer cells via miR-145-5p-mediated regulation of AKAP12. *Journal of Cellular and Molecular Medicine* **2018**, *22*, 3223-3237, doi:https://doi.org/10.1111/jcmm.13604.
186. Wang, R.; Sun, Y.; Li, L.; Niu, Y.; Lin, W.; Lin, C.; Antonarakis, E.S.; Luo, J.; Yeh, S.; Chang, C. Preclinical Study using Malat1 Small Interfering RNA or Androgen Receptor Splicing Variant 7 Degradation Enhancer ASC-J9(®) to Suppress Enzalutamide-resistant Prostate Cancer Progression. *Eur Urol* **2017**, *72*, 835-844, doi:10.1016/j.eururo.2017.04.005.
187. Li, Y.; Ji, J.; Lyu, J.; Jin, X.; He, X.; Mo, S.; Xu, H.; He, J.; Cao, Z.; Chen, X.; et al. A Novel Urine Exosomal lncRNA Assay to Improve the Detection of Prostate Cancer at Initial Biopsy: A Retrospective Multicenter Diagnostic Feasibility Study. *Cancers (Basel)* **2021**, *13*, 4075.
188. Wang, F.; Ren, S.; Chen, R.; Lu, J.; Shi, X.; Zhu, Y.; Zhang, W.; Jing, T.; Zhang, C.; Shen, J.; et al. Development and prospective multicenter evaluation of the long noncoding RNA MALAT-1 as a diagnostic urinary biomarker for prostate cancer. *Oncotarget* **2014**, *5*.
189. Ren, S.; Wang, F.; Shen, J.; Sun, Y.; Xu, W.; Lu, J.; Wei, M.; Xu, C.; Wu, C.; Zhang, Z.; et al. Long non-coding RNA metastasis associated in lung adenocarcinoma transcript 1 derived miniRNA as a novel plasma-based biomarker for diagnosing prostate cancer. *Eur J Cancer* **2013**, *49*, 2949-2959, doi:10.1016/j.ejca.2013.04.026.
190. Dai, X.; Liang, Z.; Liu, L.; Guo, K.; Xu, S.; Wang, H. Silencing of MALAT1 inhibits migration and invasion by sponging miR-1-3p in prostate cancer cells. *Mol Med Rep* **2019**, *20*, 3499-3508, doi:10.3892/mmr.2019.10602.
191. Mather, R.L.; Parolia, A.; Carson, S.E.; Venalainen, E.; Roig-Carles, D.; Jaber, M.; Chu, S.C.; Alborelli, I.; Wu, R.; Lin, D.; et al. The evolutionarily conserved long non-coding RNA LINC00261 drives neuroendocrine prostate cancer proliferation and metastasis via distinct nuclear and cytoplasmic mechanisms. *Mol Oncol* **2021**, *15*, 1921-1941, doi:10.1002/1878-0261.12954.
192. Li, Y.; Li, H.; Wei, X. Long noncoding RNA LINC00261 suppresses prostate cancer tumorigenesis through upregulation of GATA6-mediated DKK3. *Cancer Cell Int* **2020**, *20*, 474, doi:10.1186/s12935-020-01484-5.
193. Eke, I.; Bylicky, M.A.; Sandfort, V.; Chopra, S.; Martello, S.; Graves, E.E.; Coleman, C.N.; Aryankalayil, M.J. The lncRNAs LINC00261 and LINC00665 are upregulated in long-term prostate cancer adaptation after radiotherapy. *Mol Ther Nucleic Acids* **2021**, *24*, 175-187, doi:10.1016/j.omtn.2021.02.024.
